# A multi-task FP-GNN framework enables accurate prediction of selective PARP inhibitors

**DOI:** 10.3389/fphar.2022.971369

**Published:** 2022-10-11

**Authors:** Daiqiao Ai, Jingxing Wu, Hanxuan Cai, Duancheng Zhao, Yihao Chen, Jiajia Wei, Jianrong Xu, Jiquan Zhang, Ling Wang

**Affiliations:** ^1^ School of Biology and Biological Engineering, Guangdong Provincial Key Laboratory of Fermentation and Enzyme Engineering, Joint International Research Laboratory of Synthetic Biology and Medicine, Guangdong Provincial Engineering and Technology Research Center of Biopharmaceuticals, South China University of Technology, Guangzhou, China; ^2^ Department of Pharmacology and Chemical Biology, Shanghai Jiao Tong University School of Medicine, Shanghai, China; ^3^ Academy of Integrative Medicine, Shanghai University of Traditional Chinese Medicine, Shanghai, China; ^4^ Guizhou Provincial Engineering Technology Research Center for Chemical Drug R&D, College of Pharmacy, Guizhou Medical University, Guiyang, China

**Keywords:** PARP, deep learning, multi-task FP-GNN, interpretability, online webserver

## Abstract

PARP (poly ADP-ribose polymerase) family is a crucial DNA repair enzyme that responds to DNA damage, regulates apoptosis, and maintains genome stability; therefore, PARP inhibitors represent a promising therapeutic strategy for the treatment of various human diseases including COVID-19. In this study, a multi-task FP-GNN (Fingerprint and Graph Neural Networks) deep learning framework was proposed to predict the inhibitory activity of molecules against four PARP isoforms (PARP-1, PARP-2, PARP-5A, and PARP-5B). Compared with baseline predictive models based on four conventional machine learning methods such as RF, SVM, XGBoost, and LR as well as six deep learning algorithms such as DNN, Attentive FP, MPNN, GAT, GCN, and D-MPNN, the evaluation results indicate that the multi-task FP-GNN method achieves the best performance with the highest average BA, F1, and AUC values of 0.753 ± 0.033, 0.910 ± 0.045, and 0.888 ± 0.016 for the test set. In addition, Y-scrambling testing successfully verified that the model was not results of chance correlation. More importantly, the interpretability of the multi-task FP-GNN model enabled the identification of key structural fragments associated with the inhibition of each PARP isoform. To facilitate the use of the multi-task FP-GNN model in the field, an online webserver called PARPi-Predict and its local version software were created to predict whether compounds bear potential inhibitory activity against PARPs, thereby contributing to design and discover better selective PARP inhibitors.

## Introduction

Poly ADP-ribose polymerases (PARPs), an ancient protein family of 17 members, are key components of the DNA damage response in cells. Since an important study published in 2005 showed that tumor cells missing BRCA-1 or BRCA-2, critical tumor suppressor proteins involved in double-strand DNA break (DSB) repair *via* homologous recombination (HR), are more vulnerable to PARP family DNA repair enzyme inhibitors (Farmer et al., 2005), which has brought PARP inhibitors into the spotlight in the treatment of DNA repair-deficient tumors. The basic mechanism of PARP inhibitors is synthetic lethality, which refers to the occurrence of cell death caused by the simultaneous inactivation of two non-lethal genes. PARP-1, the primary target of PARP inhibitors, is involved in the repair of single-strand DNA breaks (SSBs). Nevertheless, PARP-1 suppression is not lethal since the DNA damage created by these drugs may be repaired by other DNA repair mechanisms, including HR. In the absence of BRCA1/2 and therefore a faulty HR, the PARP inhibitors-induced DNA lesions cannot be repaired, resulting in cytotoxicity (Mateo et al., 2019). Due to its inherent mechanism of action, PARP inhibitors are effective for the treatment of malignancies caused by BRCA mutations in the germline and HR deficiencies, including breast, ovarian, pancreatic, prostate, endometrial, and bile duct carcinoma cancers, particularly in patients with refractory tumors such as well-differentiated ovarian cancer and triple-negative breast cancer (TNBC).

PARP-1 catalyzes almost all intracellular PAR (de Murcia et al., 1997), which undertakes more than 90% of the PARP family functions in cells. However, intensive studies of the PARP family have revealed that other members of the PARP family play critical roles in DNA repair, gene stability, metabolism, and telomere function, making them possible therapeutic targets. For example, PARP-2 is a potential anticancer target due to its important roles in DNA repair, cell cycle regulation, metabolism, and angiogenesis (Ali et al., 2016). PARP-2 can also act as a transcriptional regulator, which plays an essential role in maintaining metabolic homeostasis (Bai et al., 2011). Furthermore, PARP-5A and PARP-5B play critical roles in the maintenance of telomeres, WNT signaling, and spindle assembly (Hsiao and Smith, 2008). Currently, four PARP inhibitors including Olaparib, Rucaparib, Niraparib, and Talazoparib have been authorized by the U.S. Food and Drug Administration (FDA) (Kim et al., 2021), which are all used for the treatment of cancers. More importantly, an increasing number of studies suggest that PARP inhibitors exhibit great potential in the treatment of non-neoplastic indications, such as ischemia (Eliasson et al., 1997), ischemia-reperfusion injury (Zingarelli et al., 1998), inflammation (Wang et al., 2013), neurological injury (Stoica et al., 2014), vascular disease (Wang et al., 2019, 2), diabetes (Masutani et al., 1999), acute lung injury (Szabo et al., 2020), as well as pulmonary fibrosis (Lucarini et al., 2017). Notably, recent studies have shown that PARP inhibitors such as CVL218, currently in Phase I clinical trials, have the potential to inhibit severe acute respiratory syndrome coronavirus 2 (SARS-CoV-2) replication (Ge et al., 2021, 19) and to combat the life-threatening sequelae of coronavirus disease 2019 (COVID-19) through multiple mechanisms (Curtin et al., 2020). However, these approved PARP inhibitors suffer certain limitations in clinical use, including toxicities (LaFargue et al., 2019), selectivity issues (known as off-target effects) (Antolín and Mestres, 2014), as well as the emergence of drug resistance (Mateo et al., 2019; Li et al., 2020). Accordingly, there is an urge to discover new PARP inhibitors for the treatment of tumor or non-tumor diseases.

Computational approaches have been used to identify or explore structure-activity relationships and atomic-level mechanisms of PARP inhibitors. For example, Hannigan et al. employed structure (docking)-based virtual screening (VS) for the discovery of five PARP-1 inhibitors with new scaffolds (Hannigan et al., 2013). In 2021, Niu and coworkers conducted an integrated VS protocol of pharmacophore modeling and molecular docking to identify a new dual tubulin/PARP-1 inhibitor (called TP-3) that displayed superior *in vitro* antiproliferative activities against human cancer cells, such as breast, liver, ovarian, and cervical cancers, and *in vivo* antitumor activity in the MDA-MB-231 xenograft model (Zheng et al., 2021). In addition, quantitative structure–activity relationships (QSAR) method was also used to study the correlation between various Benzimidazole Carboxamide (Riahi et al., 2008; Zeng et al., 2011; Sharma, 2016; Abbasi-Radmoghaddam et al., 2021) derivatives and their PARP-1 inhibitory activities, which could be utilized to predict or design better PARP-1 inhibitors. Furthermore, Kirubakaran et al. (2014) proposed the structure- and ligand-based virtual screening to explore novel PARP-5A inhibitors. Alam and coworker developed a field point based quantitative structure-activity relationship model for the identification of selective flavone ligands targeting PARP-5A and PARP-5B (Alam and Khan, 2019). These reported computational models and protocols can accelerate the exploration of the mechanism of action of specific scaffolds and the discovery of new PARP inhibitors. However, due to the high sequential homology and structural similarity of binding active sites across the PARP family, it is difficult to identify highly selective molecules for a specific PARP isoform through structure-based VS methods. Besides, current QSAR models have limited scalability because they are based on solely or few scaffolds, making it too hard to design or predict PARP inhibitors with other scaffolds. Given the significant sequential homology and structural similarity of binding active sites throughout the PARP family, it is conceivable that a multitasking model may simultaneously identify inhibitors for four isozymes, leading to improved prediction accuracy. For example, Cai et al. (2019) established a multitask deep neural network (DNN) framework for comprehensive assessment of hERG blockers, which achieved higher predictive accuracy compared to other baseline models. In 2021, Nguyen-Vo et al. (2021) developed iCYP-MFE, a computational framework for accurately predicting the inhibitory activity of molecules against five CYP isoforms (1A2, 2C9, 2C19, 2D6, and 3A4).

**FIGURE 1:**
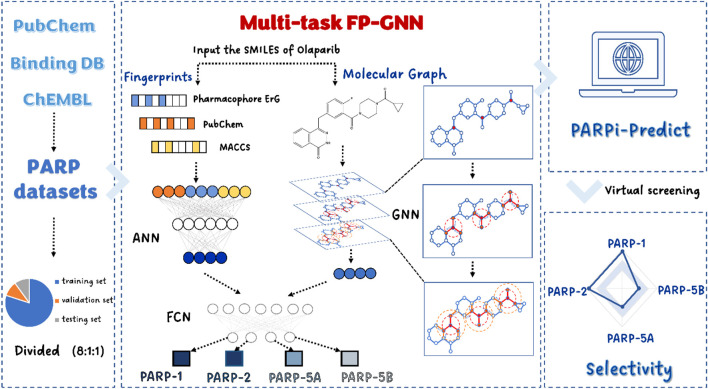
Model construction pipeline.

In the present study, we constructed an interpretable model based on a multi-task FP-GNN deep learning (DL) framework that allows for the simultaneous and accurate prediction of active molecules against four PARP subtypes (Figure 1). In addition, we exploited the interpretability of the multi-task FP-GNN model to uncover and visualize the key components of inhibitors with multiple scaffolds of PARPs inhibitory activity. Finally, our online VS platform (https://parpipredict.idruglab.cn) and python version software (https://github.com/idruglab/PARPi-Predict) could facilitate the identification and modification of selective PARP inhibitors.

## Materials and methods

### Dataset collection and preparation

The modelling datasets of PARPs inhibitors were collected from various sources such as BindingDB (Liu et al., 2007), PubChem (Kim et al., 2020), and ChEMBL (Mendez et al., 2019) databases (accessed 01 Jan 2021). The raw data were processed as the following steps: 1) kept compounds with the definitely bioassay values (assay type = B), such as IC_50_, EC_50_, *K*_d_, or *K*_i_, while compounds with no bioactivity data were discarded; 2) conversion of bioactive units (e.g., g/mL, M, and nM) to standard units in μM; 3) when a molecule has multiple biological activity data points, the average of these reported bioassay values is used as the final value; 4) duplicates and molecules with a molecular weight greater than 1,000 Da were removed; 5) each compound in the datasets was standardized to a common representation using the Python Standardizer package (https://github.com/flatkinson/standardiser) with default parameters, including removing counter-ions, solvent components, and salts, adding hydrogen atoms, and neutralizing charge by adding or subtracting atoms). To ensure the reliability and scalability of the models, the number of molecules in the modelling dataset of each PARP isoform is limited to eater than 300, resulting in a final dataset containing 4,539 unique compounds involving 5,770 bioactive data points for four PARP subtypes (i.e., PARP-1, PARP-2, PARP-5A, and PARP-5B). Finally, molecules with biological activity values (e.g., pIC_50_, pEC_50_, p*K*_i_, and p*K*_d_) ≥ 6 were labeled as actives, and *vice versa*. Each dataset was randomly partitioned into three sub-datasets: training (80%), validation (10%), and test (10%) sets. Supplementary Table S1 provides detailed information on the number of active and inactive compounds in the training, validation, and test sets.

### Multi-task FP-GNN deep learning framework and model training protocol

In the present study, a multi-task FP-GNN model (Figure 1) was developed to establish classification models for predicting active molecules against these four PARPs. Briefly, the FP-GNN algorithm simultaneously learns molecular graph information and fixed prior molecular fingerprints information to better predict molecular properties, including molecular physicochemical properties, biological activities, and ADMET properties. Graph-based module of FP-GNN model employs a spatial graph neural network (GNN) with attention mechanism to acquire structural information in molecular graphs, while the fingerprint-based network module of FP-GNN uses an artificial neural network (ANN) to learn information from two substructure-based molecular fingerprints (PubChem FP and MACCS FP) as well as a pharmacophore-based fingerprint (Pharmacophore ErG FP). FP-GNN finally uses fully convolutional networks (FCN) to fuse the features from both GNN and FPN, and then outputs the prediction results of molecular properties. FP-GNN DL algorithm achieves state-of-the-art (SOTA) performance on multiple molecular property prediction tasks (Cai et al., 2022), and is freely available at (https://github.com/idrugLab/FP-GNN). However, most datasets in drug discovery have substantial connections between subtasks. Data association information between subtasks (e.g., various PARP subtypes) will be lost if just a single task model is employed for the training test. Therefore, we proposed the multi-task FP-GNN model to prevent data loss from subtasks. The multi-task FP-GNN adopts the parameter sharing multi-task learning method, inherits the molecular graph and molecular fingerprints modules from the single task model, and extends the fusion module into a multi-task output module. During training the multi-task FP-GNN model in this study, Bayesian optimization method was used to optimize the following hyperparameters: Dropout (0, 0.05, 0.1, 0.15, 0.2, 0.25, 0.3, 0.35, 0.4, 0.45, 0.5, 0.55, 0.6), dropout gat (0, 0.05, 0.1, 0.15, 0.2, 0.25, 0.3, 0.35, 0.4, 0.45, 0.5, 0.55, 0.6), dim (300, 350, 400, 450, 500, 550, 600), gat scale (0.2, 0.3, 0.4, 0.5, 0.6, 0.7, 0.8), nheads (2, 3, 4, 5, 6, 7, 8), nhid (40, 45, 50, 55, 60, 65, 70, 75, 80). Among these hyperparameters, dropout and dropout gat represent the dropout rates of fingerprint networks and graph neural networks, respectively. The number of multi-head attentions and the hidden size of attentions are controlled by nheads and nhid in the attention mechanism. The hidden size of fingerprint networks is affected by hyperparameter dim. Gat scale is used to determine the ratio of GNN and FPN in FP-GNN for tunning their weights. To eliminate randomness and ensure the generalization capacity of our models, we performed 10 independent runs with different seeds to train and evaluate the multi-task FP-GNN models and then computed the average values of evaluation metrics as the results.

### The baseline machine learning and deep learning algorithms

To further fairly compare the multi-task FP-GNN model in the PARPs inhibitors prediction tasks, we established predictive models using four conventional machine learning (CML) algorithms, i.e., random forests (RF) (Breiman, 2001), support vector machine (SVM) (Cortes and Vapnik, 1995), extreme gradient boosting (XGBoost) (Chen and Guestrin, 2016), and logistic regression (LR) (Davis and Offord, 1997) and six DL algorithms such as deep neural networks (DNN) (Durstewitz et al., 2019), Attentive FP (Xiong et al., 2020), D-MPNN (Chemprop) (Petras et al., 2021), graph attention network (GAT) (Velickovic et al., 2018), graph convolutional networks (GCN) (Duvenaud et al., 2015), and message passing neural networks (MPNN) (Gilmer et al., 2017). A brief introduction to these CML and DL methods can be found elsewhere (Dreiseitl and Ohno-Machado, 2002; Wang et al., 2014; Wu et al., 2017; He et al., 2021). In this study, two commonly used molecular fingerprints including MACCS keys (Durant et al., 2002) (MACCS, 166 bits) and Morgan fingerprint (Rogers and Hahn, 2010) (termed ECFP_4, 1,024 bits) were employed to construct CML and DNN models. Other DL methods (GAT, Chemprop, Attentive FP, GCN, and MPNN) implemented in DeepChem software used molecular graphs as input features. MolGraphConvFeatureizer was used to produce molecular graphs for the GAT, Attentive FP, and MPNN models, while the convmolfeatureizer module was used to calculate molecular graphs for the GCN model using (Duvenaud et al., 2015). All Fingerprints and graph features were generated based on the smiles of compounds using RDKit software.

The RF, SVM, and LR models were created using the scikit-learn python package (https://github.com/scikit-learn/scikit-learn, version: 0.24.1) (Pedregosa et al., 2018); the XGBoost models were developed using the XGBoost python package (https://github.com/dmlc/xgboost, version: 1.3.3) (Chen and Guestrin, 2016); and other graph-based DL models were constructed using the DeepChem Python package (https://deepchem.io/). All these CML and DL models, as well as FP-GNN models presented here were trained on the CPU (Intel(R) Xeon(R) Silver 4216 CPU at 2.10 GHz) and GPU (NVIDIA Corporation GV100GL [Tesla V100 PCIe 32 GB]).

### Performance evaluation of models

The performance of the multi-task FP-GNN model as well as the baseline CML and DL models is evaluated using the following metrics, including specificity (SP/TNR), sensitivity (SE/TPR/Recall), accuracy (ACC), F1-measure (F1 score), Matthews correlation coefficient (MCC), the area under the receiver operating characteristic (AUC), and balanced accuracy (BA). The AUC is defined as the area under the receiver operating characteristic curve (ROC) that plots TPR vs. FPR at different classification thresholds. Six evaluation metrics are defined as follows:

$$SP=\frac{TN}{TN+FP}$$ (1)

$$SE=\frac{TP}{TP+FN}$$ (2)

$$ACC=\frac{TP+TN}{TP+TN+FP+FN}$$ (3)

$$F1=\frac{2\times Precision\times Recall}{Precision+Recall}=\frac{2\times TP}{2\times TP+FN+FP}$$ (4)

$$MCC=\frac{TP\times TN-FN\times FP}{\sqrt{\left(TP+FN\right)\times \left(TP+FP\right)\times \left(TN+FN\right)\times \left(TN+FP\right)}}$$ (5)

$$BA=\frac{TPR+TNR}{2}=\frac{SE+SP}{2}$$ (6)

The number of true positives, true negatives, false positives, and false negatives is represented as TP, TN, FP, and FN, respectively. The AUC metric was computed using the scikit-learn python package. All models were optimized and selected based on the BA value. The best-performing multi-task FP-GNN model was saved and unitlized for the development of the online VS platform (called PARPi-Predict) and python version large-scale VS software.

### Model applicability domain

Typically, the Organization for Economic Cooperation and Construction (OECD) recommends the establishment of an applicability domain (AD) for QSAR models, which allows users to evaluate uncertainty in the prediction of a chemical based on how similar it is to the training compounds used in model development. In this work, we used a structural similarity-based approach called the Euclidean distance-based method (DM) for AD analysis. Morgan fingerprints is used to depict the chemical structures. This procedure will eventually yield a distance threshold (*D*_*T*_) that can be used to assess whether the chemical is inside the AD of the model. The detailed *D*_*T*_ is expressed as follows:

$$D_{T}=d_{ave}+Z\times \theta$$ (7)

Where *d*_*ave*_ is the average Euclidean distance between each compound in the training set and its nearest k compounds, *θ* is the corresponding standard deviation, and *Z* is an optional parameter representing the significance level. First, we calculate the fingerprints of the test set and the training set by RDKit software, and then we calculate the average of the Euclidean distance. *d*_*ave*_ and *θ* are obtained from the Euclidean distances of the k nearest neighbors for each molecule in the training set. Finally, the Euclidean distance between each molecule in the test set and the nearest neighbor molecule in the training set is calculated. If the distance exceeds the threshold of *D*_*T*_, the compound is considered to be outside the domain (OD). Otherwise, it has fallen into the domain (ID). Herein, we used the test set to find suitable parameters k and *Z*, and then determined the threshold of the AD of the model (Horvath et al., 2010).

## Results

### Dataset analysis and model construction

**FIGURE 2:**
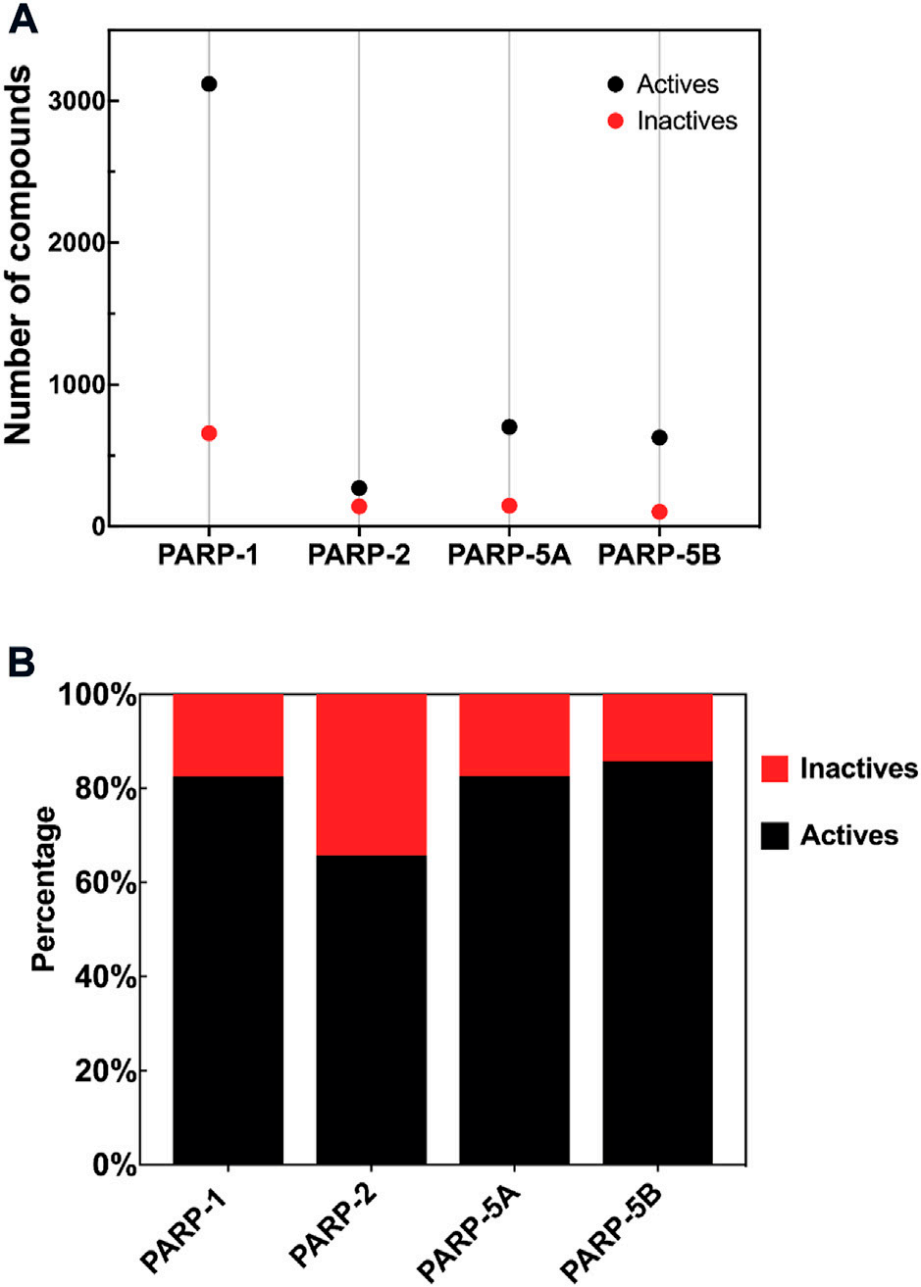
**(A)** Number of compounds in each PARP isoform dataset. **(B)** Percentage of active and inactive compounds in each PARP isoform.

According to the above-predefined criteria, four PARP isoforms (PARP-1, PARP-2, PARP-5A, and PARP-5B, Figure 2A) datasets were collected and filtered from the PubChem, ChEMBL, and BindingDB databases. Details on the four PARP isoforms and their corresponding target associated compound datasets are listed in Table 1. 4,539 unique compounds involved 5,770 bioassay data points for these four PARP subtypes. Among these target-compound associations, 4,720 compounds were labeled as actives (positives) and 1,050 compounds were labeled as inactives (negatives). As shown in Figure 2B, the active compounds accounted for between 65.78% and 85.79% in the four PARP isoforms, implying that there is a data imbalance in the PARP datasets. Due to the natural, we did not add any theoretical decoys to deliberately balance the PARP modelling datasets in this study (Wang et al., 2017), although they may not be the best.

**TABLE 1:** The modelling datasets of PARPs inhibitors.

Target	UniProt ID	No. of compounds	No. of scaffolds	Scaffolds/compounds (%)
PARP-1	P09874	3,777	663	17.55
PARP-2	Q9UGN5	412	110	26.70
PARP-5A	O95271	849	175	20.61
PARP-5B	Q9H2K2	732	159	21.72

**FIGURE 3:**
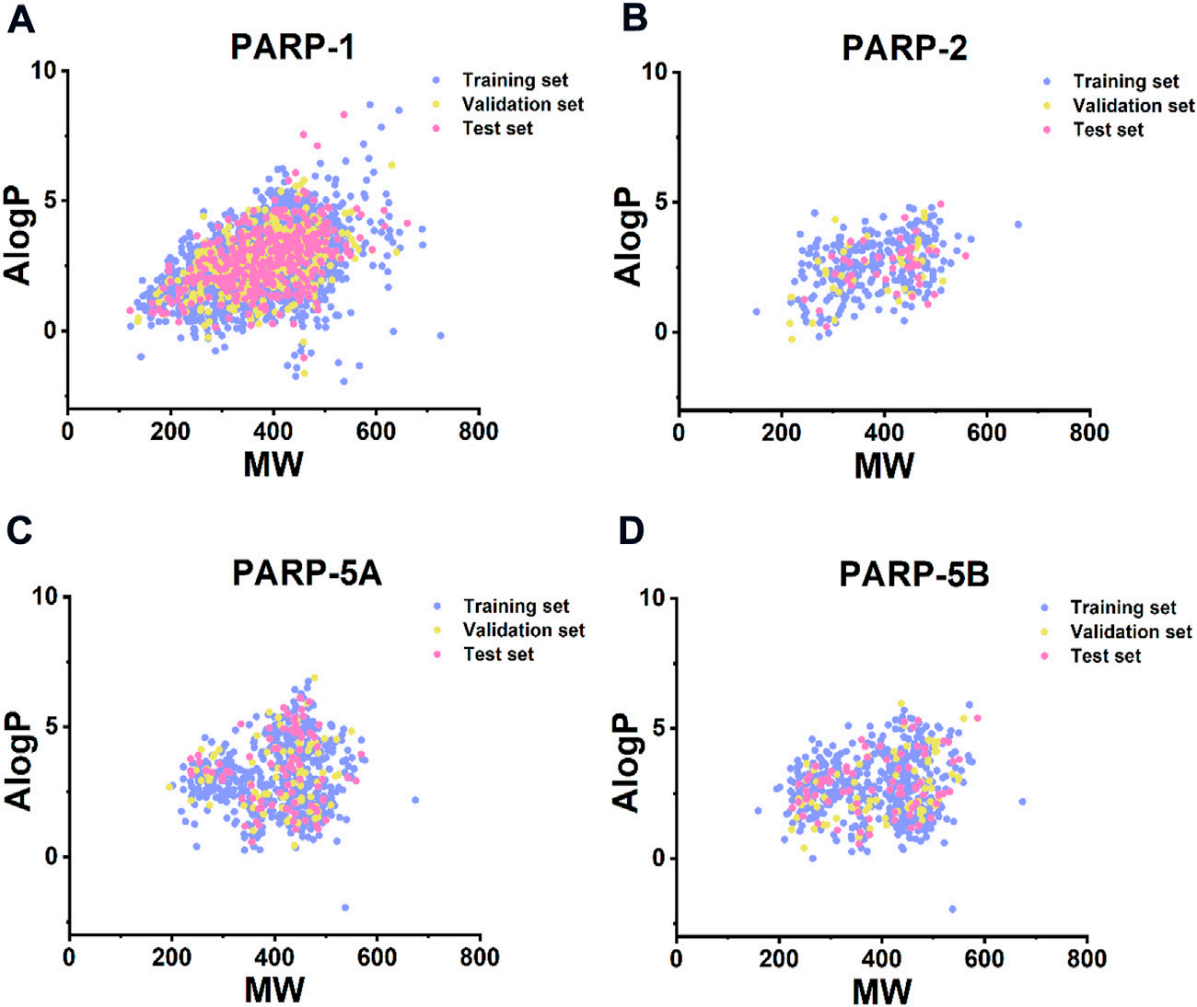
Chemical space analysis of the compounds in **(A)** PARP-1 **(B)** PARP-2, **(C)** PARP-5A, and **(D)** PARP-5B datasets. Chemical space was defined using molecular weight (MW, *X-axis*) and AlogP (*Y-axis*). MW and AlogP were computed using RDKit software.

The structural diversity and large chemical space of the molecules in the modelling datasets can help to build accurate and robust predictive models (Wang et al., 2016b; Luo et al., 2019; Guo et al., 2020). According to Bemis Murcko scaffold analysis (Bemis and Murcko, 1996), the proportion of the scaffolds in the PARP inhibitors modelling datasets ranged from 17.55% to 26.70% (Table 1), indicating considerable structural diverse of compounds within each PARP subtype. Furthermore, compounds in the training, validation, and test sets have a wide range of molecular weight (MW, 121.139–725.683) and AlogP (−1.946–8.700) (Figure 3), indicating that the compounds in the modelling datasets have a broad chemical space.

### Evaluation results of fingerprint-based predictive models

40 models were constructed based on Morgan and MACCS fingerprints using four CML algorithms (RF, SVM, XGBoost, and LR) and one DL DNN method. All models are represented by a combination of an algorithm and a given molecular representation (e.g., DNN:Morgan). Detailed hyperparameter optimization and performance results of these fingerprint-based predictive models are provided in Supplementary Tables S2–S4.

**FIGURE 4:**
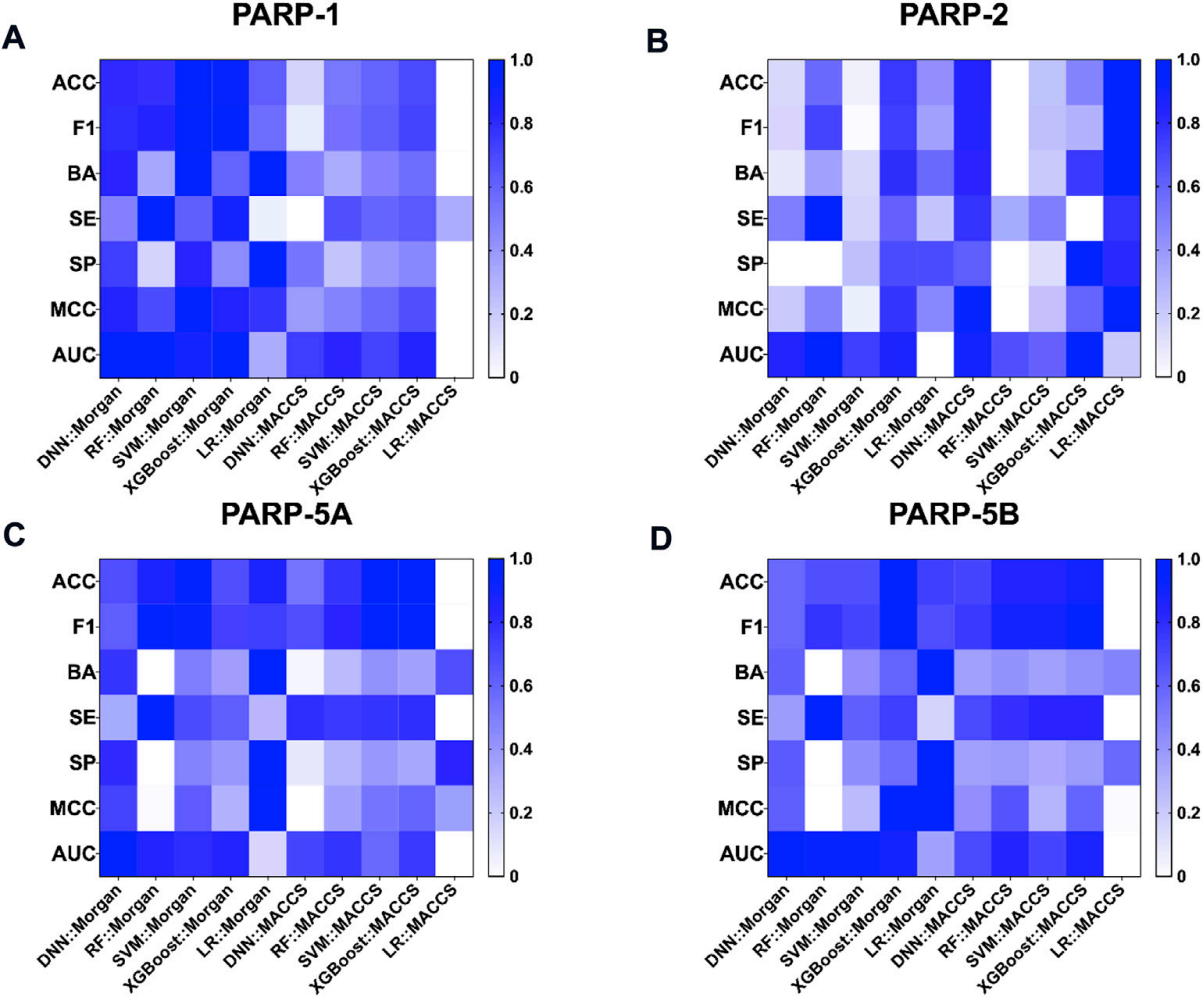
Performance of fingerprint-based prediction models on the test sets of PARP-1 **(A)**, PARP-2 **(B)**, PARP-5A **(C)**, and PARP-5B **(D)**.

As shown in Table 2, the fingerprint-based models achieved better performance on the test set for each PARP isoform, with average values of BA, F1 score and AUC greater than 0.65, 0.85 and 0.65, respectively. Taking the average BA value as the main evaluation metric to take the imbalance of the data into account, the Morgan-based models showed slightly better overall predictive performance compared with the MACCS-based models. Among them, the LR:Morgan models performed the best with the highest average values of BA (0.735 ± 0.047), SP (0.554 ± 0.095), and MCC (0.493 ± 0.085). Meanwhile, the LR:Morgan models ranked first in three of the four PARP subtypes (PARP-1, PARP-5A, and PARP-5B, Supplementary Table S5; Figure 4). Furthermore, the DNN:Morgan models also achieved very competitive performance, with the second-ranked mean BA value, as well as the highest mean AUC value. In addition, Supplementary Figure S1 illustrates that there is no significant difference in the predictive performance of the models generated by different ML algorithms combined with the same fingerprint.

**TABLE 2:** The overall predictive performance of fingerprint-based models for the test sets.

Model	Test set						
	ACC^a^	F1^b^	BA^c^	SE^d^	SP^e^	MCC^f^	AUC^g^
DNN::Morgan	0.841 ± 0.059	0.899 ± 0.049	0.703 ± 0.057	0.930 ± 0.034	0.476 ± 0.109	0.468 ± 0.106	**0.873 ± 0.043**
XGBoost::Morgan	**0.854 ± 0.049**	**0.909 ± 0.042**	0.700 ± 0.069	0.947 ± 0.039	0.454 ± 0.155	0.486 ± 0.120	0.861 ± 0.050
SVM::Morgan	0.844 ± 0.063	0.901 ± 0.054	0.692 ± 0.074	0.936 ± 0.051	0.448 ± 0.163	0.453 ± 0.129	0.854 ± 0.050
RF::Morgan	0.849 ± 0.049	0.908 ± 0.041	0.655 ± 0.095	**0.965 ± 0.034**	0.344 ± 0.212	0.429 ± 0.142	0.870 ± 0.051
LR::Morgan	0.846 ± 0.052	0.901 ± 0.045	**0.735 ± 0.047**	0.917 ± 0.037	**0.554 ± 0.095**	**0.493 ± 0.085**	0.735 ± 0.047
Average (Morgan)	0.847	0.904	0.697	0.939	0.455	0.466	0.839
XGBoost::MACCS	0.850 ± 0.053	0.904 ± 0.049	0.691 ± 0.075	0.939 ± 0.061	0.443 ± 0.189	0.470 ± 0.104	0.853 ± 0.046
DNN::MACCS	0.846 ± 0.038	0.904 ± 0.035	0.681 ± 0.087	0.944 ± 0.032	0.417 ± 0.199	0.448 ± 0.127	0.832 ± 0.041
SVM::MACCS	0.846 ± 0.057	0.903 ± 0.049	0.680 ± 0.067	0.946 ± 0.044	0.414 ± 0.157	0.444 ± 0.107	0.817 ± 0.047
RF::MACCS	0.841 ± 0.062	0.900 ± 0.053	0.673 ± 0.063	0.943 ± 0.049	0.403 ± 0.148	0.442 ± 0.099	0.841 ± 0.044
LR::MACCS	0.835 ± 0.031	0.897 ± 0.028	0.695 ± 0.058	0.920 ± 0.023	0.470 ± 0.117	0.435 ± 0.116	0.695 ± 0.058
Average (MACCS)	0.844	0.902	0.684	0.938	0.429	0.448	0.808

a ACC, accuracy.

b F1, F1-measure.

c BA, balanced accuracy.

d SE, sensitivity.

e SP, specificity.

f MCC, matthews correlation coefficient.

g AUC, the area under receiver operating characteristic.

Bold font illustrates the models that outperformed all other models.

### Evaluation results of graph-based DL models

Recently, GNN and variants, which utilize molecules as natural graph structure data, have been developed and widely used in various drug discovery related tasks. GNN models and their variations (e.g., GAT, GCN, MPNN, Attentive FP, and Chemprop) have been reported to achieve SOTA performance in several molecular property prediction tasks (Wu et al., 2017; Yang et al., 2019; Xiong et al., 2020).

**FIGURE 5:**
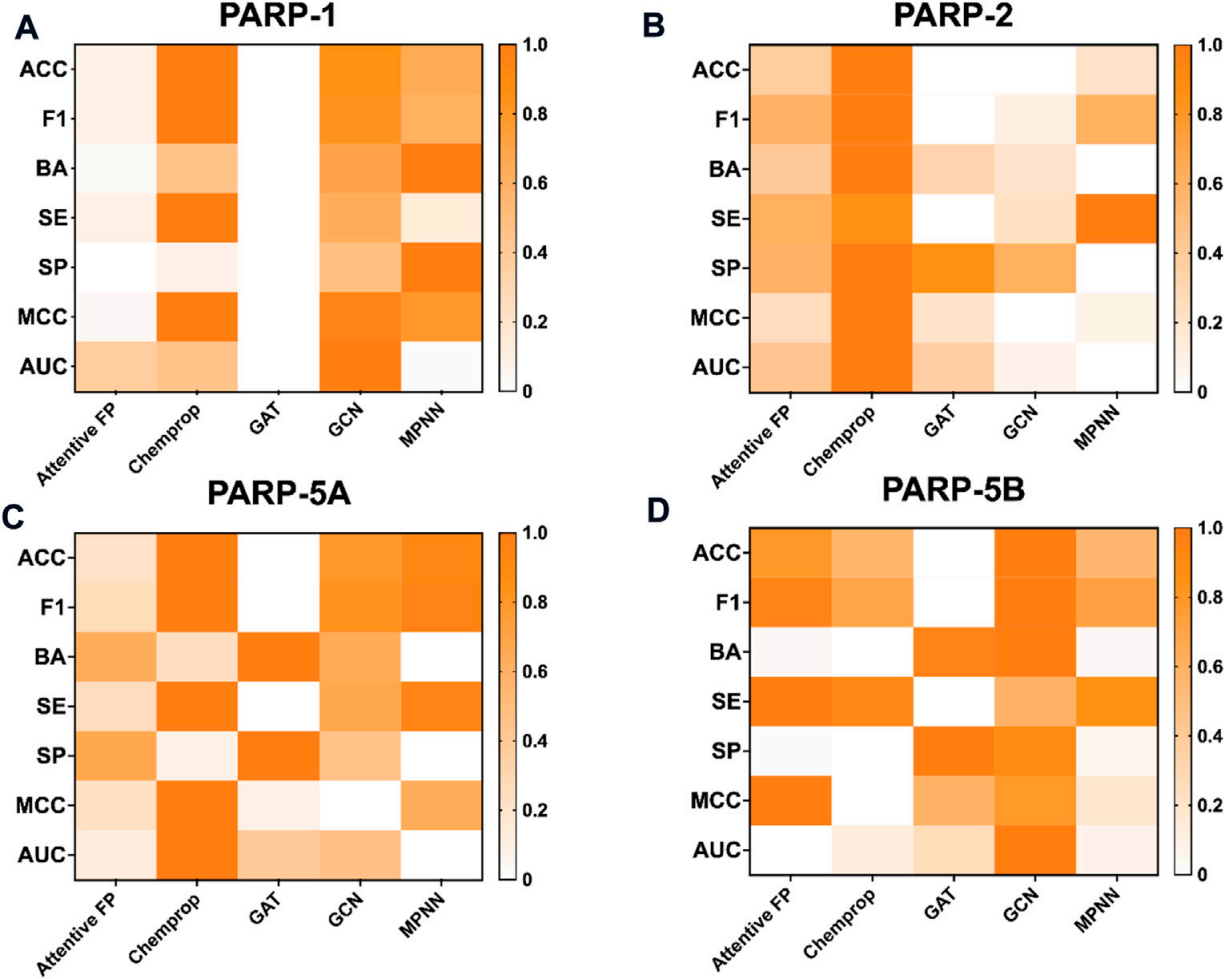
Performance of graph-based DL models on the test sets of PARP-1 **(A)**, PARP-2 **(B)**, PARP-5A **(C)**, and PARP-5B **(D)**.

We therefore used five DL methods (GAT, GCN, MPNN, Attentive FP, and Chemprop) to create 20 graph-based DL models for four PARP isoforms. Supplementary Tables S2, S6 provide the detailed hyperparameter setting and performance of the graph-based DL models. As shown in Table 3, compared with other GNN approaches, GAT had a relatively higher average BA value (0.673 ± 0.066) for the test sets, but relatively poor other evaluation metrics. Meanwhile, Chemprop (D-MPNN) achieved the highest F1, AUC, ACC, and MCC values overall. Similar to fingerprint-based models, Figure 5; Supplementary Table S6 show that no GNN method can achieve the optimal prediction results on all or most PARP subtypes. For example, when the BA value is used as the final evaluation metric to select the best-performing model, the MPNN, Chemprop, GAT, and GCN models performed the best on PARP-1, PARP-2, PARP-5A, and PARP-5B, respectively. In addition, it is clear that the graph-based DL models are far inferior to the fingerprint-based ML models. The inherent self-learning mechanism of graph-based DL methods suffers from insufficient PARP modelling datasets, especially for PARP-2, PARP-5A, and PARP-5B isoforms (Table 1), limiting their performance.

**TABLE 3:** The overall predictive performance of graph-based DL models for the test sets (sorted by BA value).

Methods	Test set						
	ACC^a^	F1^b^	BA^c^	SE^d^	SP^e^	MCC^f^	AUC^g^
GAT	0.801 ± 0.090	0.861 ± 0.086	**0.673** ± **0.066**	0.871 ± 0.096	**0.475** ± **0.124**	0.399 ± 0.129	0.803 ± 0.051
GCN	0.822 ± 0.096	0.881 ± 0.087	0.667 ± 0.078	0.914 ± 0.091	0.420 ± 0.160	0.398 ± 0.153	0.823 ± 0.079
Chemprop	**0.848** ± **0.047**	**0.904** ± **0.045**	0.664 ± 0.115	0.955 ± 0.053	0.372 ± 0.269	**0.462** ± **0.144**	**0.832** ± **0.052**
Attentive FP	0.819 ± 0.077	0.883 ± 0.064	0.649 ± 0.093	0.920 ± 0.067	0.378 ± 0.208	0.421 ± 0.120	0.799 ± 0.065
MPNN	0.826 ± 0.083	0.893 ± 0.063	0.630 ± 0.106	**0.956** ± **0.038**	0.303 ± 0.226	0.404 ± 0.141	0.783 ± 0.065

a ACC, accuracy.

b F1, F1-measure.

c BA, balanced accuracy.

d SE, sensitivity.

e SP, specificity.

f MCC, matthews correlation coefficient.

g AUC, the area under receiver operating characteristic.

Bold font illustrates the models that outperformed all other models.

### Evaluation results of multi-task FP-GNN model

FP-GNN, a new DL architecture created in our Lab, can operate over a hybrid molecular representation of molecular graphs and fingerprints to enhance molecular property prediction (Cai et al., 2022). However, there may be correlations between subtasks of datasets in the field of drug discovery and development, such as PARP subtypes. To solve this problem, we proposed a multi-task FP-GNN model to lessen the risk of loss of data association information between sub-tasks, with the aim of further improving performance on the modelling datasets of four PARP isoforms. The detailed hyperparameter setting and performance of the multi-task FP-GNN and the corresponding single-task FP-GNN models are summarized in Supplementary Tables S2, S7, respectively.

**FIGURE 6:**
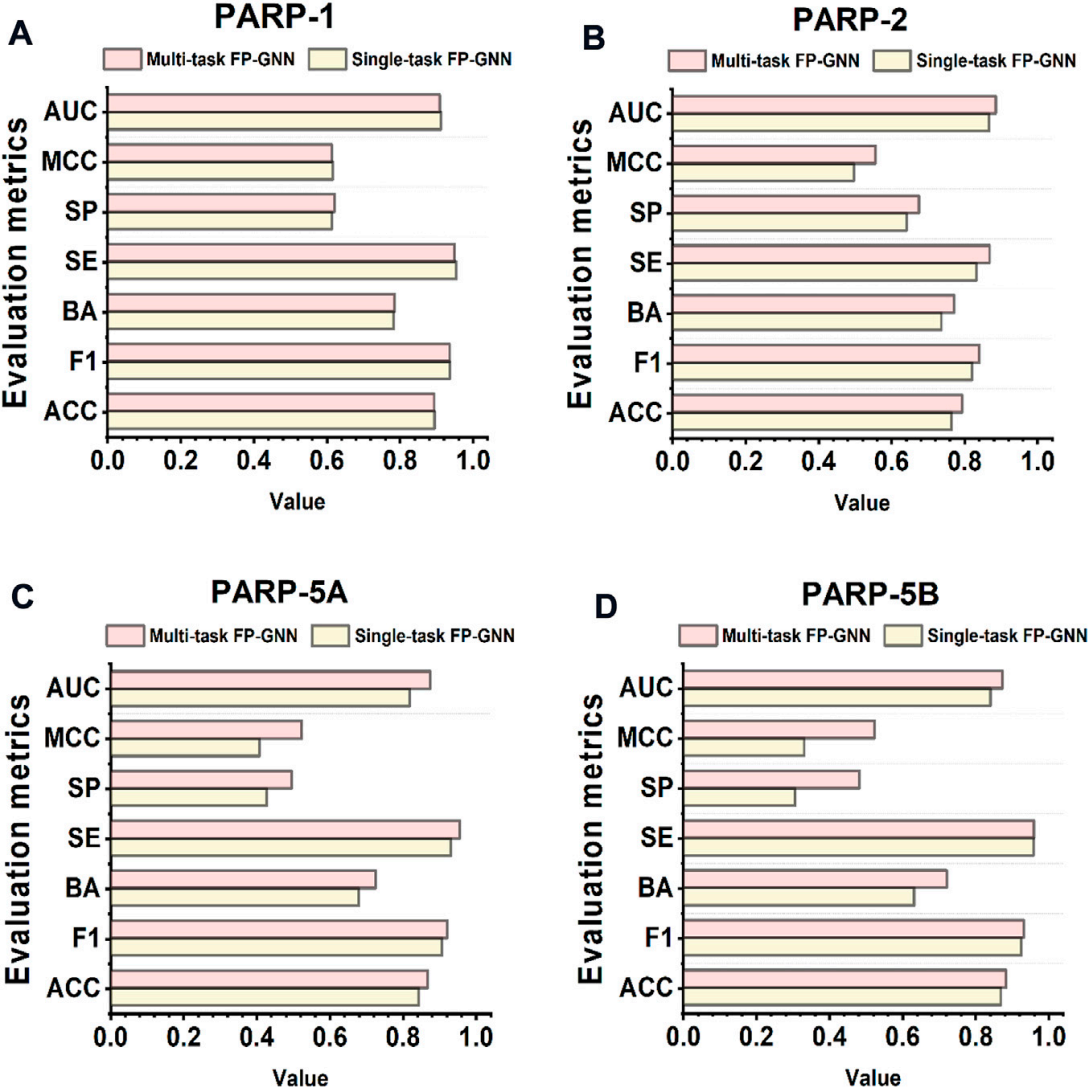
Performance of single-task FP-GNN and multi-task FP-GNN models on the test sets of PARP-1 **(A)**, PARP-2 **(B)**, PARP-5A **(C)**, and PARP-5B **(D)**.

**FIGURE 7:**
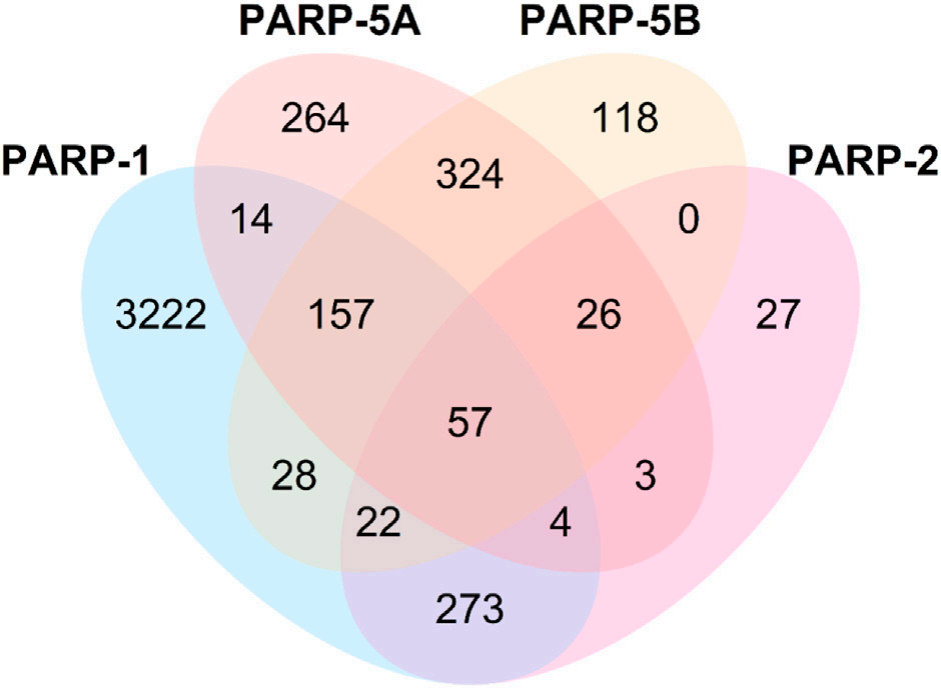
Common molecules in four PARP isoforms.

As shown in Table 4, the average values of seven evaluation metrics of the multi-task FP-GNN model for the test sets are significantly higher than that of the single-task FP-GNN model, demonstrating that the multi-task FP-GNN model outperform the single-task FP-GNN model overall in predicting the inhibitory activity of molecules against four PARP targets. Specifically, the multi-FP-GNN model can simultaneously improve the prediction performance of inhibitors of four PARP subtypes (Figure 6), especially for PARP-2 (Figure 6B), PARP-5A (Figure 6C), and PARP-5B (Figure 6D). Data point distribution analysis further revealed that four PARP isoforms share a large number of common molecular entities (Figure 7), which explains the outstanding performance of the multi-task FP-GNN model.

**TABLE 4:** The overall predictive performance of the single-task FP-GNN and multi-task FP-GNN model.

Model	Test set						
	ACC^a^	F1^b^	BA^c^	SE^d^	SP^e^	MCC^f^	AUC^g^
Single-task FP-GNN	0.845 ± 0.056	0.899 ± 0.053	0.710 ± 0.066	0.921 ± 0.059	0.498 ± 0.159	0.464 ± 0.123	0.862 ± 0.040
Multi-task FP-GNN	**0.862** ± **0.046**	**0.910** ± **0.045**	**0.753** ± **0.033**	**0.936** ± **0.044**	**0.570** ± **0.095**	**0.556** ± **0.042**	**0.888** ± **0.016**

a ACC, accuracy.

b F1, F1-measure.

c BA, balanced accuracy.

d SE, sensitivity.

e SP, specificity.

f MCC, matthews correlation coefficient.

g AUC, the area under receiver operating characteristic.

Bold font illustrates the models that outperformed all other models.

### Comparison performance of fingerprint-based, graph-based, FP-GNN, and multi-task FP-GNN models

**FIGURE 8:**
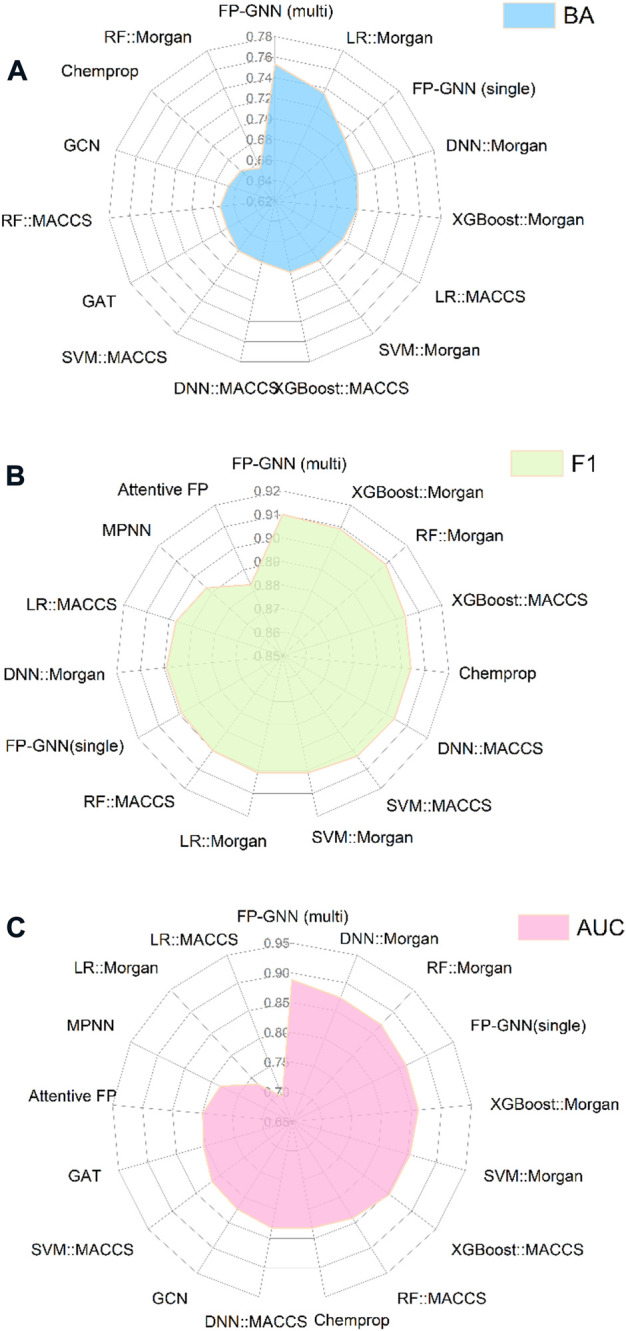
Comparison performance of all established predictive models. **(A)**, **(B)**, and **(C)** represent the average BA, F1, and AUC values of the test sets.

The comprehensive evaluation results of the above-established prediction models indicated that the multi-task FP-GNN method achieved the best performance, with the highest average BA, F1, and AUC values of 0.753 ± 0.033, 0.910 ± 0.045, and 0.888 ± 0.016 for the test sets (Figure 8; Supplementary Table S8). The detailed multi-task FP-GNN prediction model for each PARP isoform is provided in Table 5. Such results indicate that the multi-task FP-GNN algorithm shows superiority in predicting the biological activity of molecules. Y-scrambling test was performed to prove that the results of the multi-task FP-GNN model were not due to a chance connection. Supplementary Figure S2 illustrates that the BA, F1, and AUC values of the multi-task FP-GNN model were significantly higher than those of the Y-scrambled models, confirming that the results were not chance correlations.

**TABLE 5:** The detailed performance of the multi-task FP-GNN model for each PARP isoform.

Target	Model	Test set						
		ACC^a^	F1^b^	BA^c^	SE^d^	SP^e^	MCC^f^	AUC^g^
PARP-1	Multi-task FP-GNN	0.897	0.938	0.788	0.952	0.623	0.615	0.911
PARP-2	Multi-task FP-GNN	0.795	0.843	0.774	0.871	0.677	0.558	0.888
PARP-5A	Multi-task FP-GNN	0.871	0.923	0.728	0.958	0.497	0.525	0.877
PARP-5B	Multi-task FP-GNN	0.886	0.934	0.723	0.962	0.483	0.525	0.876

^a^ACC, accuracy.

b F1, F1-measure.

c BA, balanced accuracy.

d SE, sensitivity.

e SP, specificity.

f MCC, matthews correlation coefficient.

g AUC, the area under receiver operating characteristic.

### Interpretation of the multi-task FP-GNN model

**FIGURE 9:**
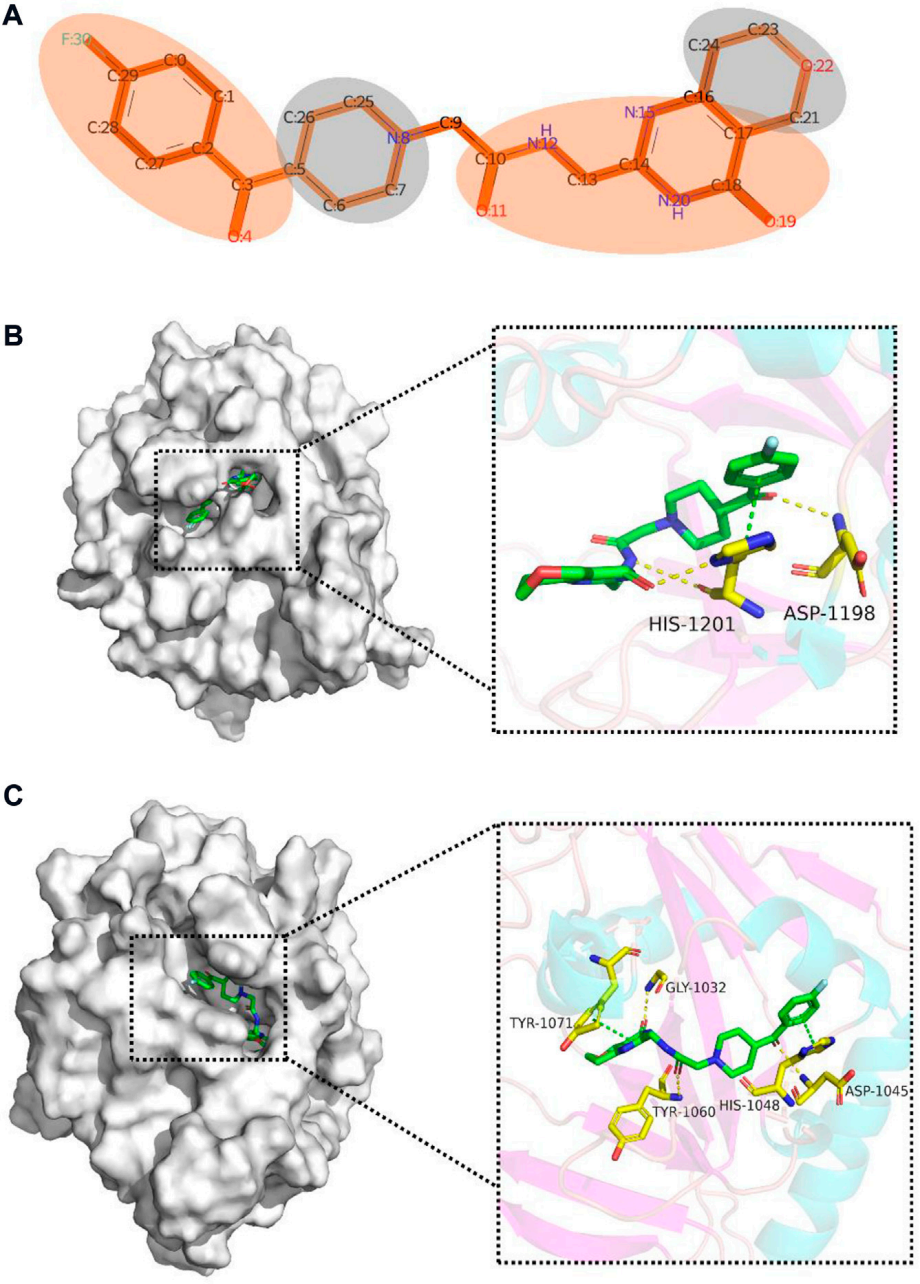
Interpretative analysis of the predicted results of the active compound (CHEMBL2419697) with high selectivity for PARP-5A and PARP-5B in the multi-task FP-GNN model. **(A)** Mapping the attention coefficients to the active molecule through the GNN module in the multi-task FP-GNN architecture to highlight substructures/chemical fragments that contribute significantly to the predicted outcomes. **(B)** and **(C)** represent the predicted binding modes of the molecule to PARP-5A (PDB ID: 3UDD) (Glide-XP docking score: −8.004 kcal/mol) and PARP-5B (PDB ID: 7CE4) (Glide-XP docking score: −8.602 kcal/mol), respectively. π-π interactions are indicated by green dotted lines, and hydrogen bonds are depicted by yellow dotted lines. The binding modes were predicted using Glide-XP docking and the figures were generated using PyMOL software (https://pymol.org/2/).

To gain insight into the multi-task FP-GNN model for the prediction of PARP inhibitors, we performed an interpretability analysis of its GNN and FPN modules. Taking a selective PARP-5A/5B inhibitor (CHEMBL2419697, Figure 9A) as an example (PARP-5A, IC_50_ = 0.0645 μM; PARP-5B, IC_50_ = 0.023 μM; PARP-1, IC_50_ = 10.4 μM; PARP-2, IC_50_ = 3.15 μM) (Shultz et al., 2013), the multi-task FP-GNN architecture can calculate the attention of adjacent atoms and map it to the bonds connected to the atoms. A higher attention coefficient for a given molecule represents a greater contribution of chemical fragments to the prediction of molecular biological activity. The portions of the molecule colored more darkly were more relevant in predicting whether the molecule could inhibit PARP-5A activity, whereas the light-colored parts are less critical for the inhibition of PARP-5A activity (Figure 9A). To further analyze the mechanism of inhibitory effects of these chemical fragments on PARP targets, molecular docking was used to investigate the binding modes of the molecule with PARP-5A, PARP-5B, PARP-1, and PARP-2. The chemical structure of compound CHEMBL2419697 was chemically standardized (including ionizing at the pH range from −2.0 to 7.0 using Epik, choosing desalt and generate tautomers option, retaining specified chiralities, and optimization based on OPLS_2005 force field) by means of the LigPrep module in Maestro (version 9.4, Schrödinger). The PARPs proteins were manipulated using the “Protein Preparation Wizard” workflow in Maestro with default parameters, including the removal of all water molecules, protonation, and optimization based on the OPLS_2005 force field (Wang et al., 2016a). Re-docking studies demonstrate that the Glide docking method is qualified for docking small molecules to the PARP proteins (Supplementary Table S9). Previous studies have shown that compounds form interactions with key residues, such as Gly863 and Ser904 in PARP-1 (Tomassi et al., 2020), Gly429 and Ser470 in PARP-2 (Papeo et al., 2015), Asp1198 and His1201 in PARP-5A (Shultz et al., 2012), as well as Asp1045, Tyr1071, Gly1032, His1048, and Tyr1060 in PARP-5B (Karlberg et al., 2010; Shirai et al., 2019; Kinosada et al., 2021), which are necessary to maintain their inhibitory activity against PARPs. As shown in Figure 9B, the molecule can form three H-bonds with Asp1198 and His1201 as well as a π-π interaction with His1201 in the binding pocket of PARP-5A. Moreover, the 4-fluorophenyl moiety can form hydrophobic interactions with the surrounding hydrophobic amino acids such as Ile1192, Ala1191, and Phe1188 (Supplementary Figure S3A). Meanwhile, it also forms three H-bond interactions with Gly1032, Asp1045, and Tyr1060 as well as π-π interactions with Tyr1071 and His1048 in PARP-5B (Figure 9C). Furthermore, the 4-fluorophenyl moiety can form hydrophobic interactions with the surrounding hydrophobic amino acids such as Ile1039, Ala1038, and Phe1035 (Supplementary Figure S3B). However, Supplementary Figure S4 shows that the molecule fails to form visible interactions with key residues of PARP-1 (e.g., Gly863 and Ser904) and PARP-2 (e.g., Gly429 and Ser470). Obviously, the chemical fragments in red (Figure 9A) interact with key amino acids of PARP-5A/5B (Figures 9B,C), demonstrating that the high attention highlighted in the red portion from the multi-task FP-GNN model was consistent with the binding modes analysis results.

In addition to the GNN module, we investigated the interpretation of the FPN module on PARP modelling datasets. The 20 most significant bits are shown in Supplementary Table S10, which may facilitate in the design and optimization of new PARP-selective inhibitors. Among these top crucial bits, there are 15 bits coming from the Pharmacophore ErG FP. Such results illustrate that the Pharmacophore ErG FP plays an important role in the prediction of PARP inhibitors (Supplementary Table S10). For clarity, we simplified the original structure of the active molecule (CHEMBL2419697, Figure 9A) according to the concept of ErG (Stiefl et al., 2006) (Supplementary Figure S5). Supplementary Figure S6 shows that the substructures represented by the 4th, 5th, 12th, 13th, 14th, and 19th bits are important components of this active molecule. Furthermore, the chemical fragments of interest in the FPN module (Supplementary Figure S6) interact with key residues of PARP-5A/5B (Figures 9B,C). Therefore, our multi-task model can capture the important chemical fragments from the FPN module, which can intuitively explain the prediction results of the model.

### Model AD analysis

We experimented with various k and *Z* values and eventually obtained the corresponding number of OD compounds (Supplementary Table S11). It can be seen that the subsequent increase in *Z* values and the stay in k resulted in a continual drop in compounds outside the AD. Posteriorly, the multi-task FP-GNN model was used to predict the ID and OD chemicals in the test set at various k and *Z* values, and the detailed performance of PARP dataset is presented in Supplementary Table S12. We noticed that when k = 2, *Z* = 0.4, and k = 3, *Z* = 0.2, the overall evaluation metrics of the model were improved, and it was able to distinguish ID and OD compounds of the PARP dataset to the maximum extent (the predictive performance of ID compounds was significantly better than that of OD compounds). These findings indicated that our defined AD for PARP dataset is suitable for the proposed multi-task FP-GNN model. Notably, the multi-task FP-GNN model can predict not only ID compounds but also OD compounds well in the prediction of PARP inhibitors (Supplementary Table S12). Undoubtedly, this phenomenon illustrates the advantages of a multi-task learning strategy that exploits the linkages between various subtasks while recognizing their distinctions. Even if a compound lacks information from a task, it can potentially increase the fault tolerance of the model by identifying missing data *via* eavesdropping or hint mechanism.

### Webserver construction and use

**FIGURE 10:**
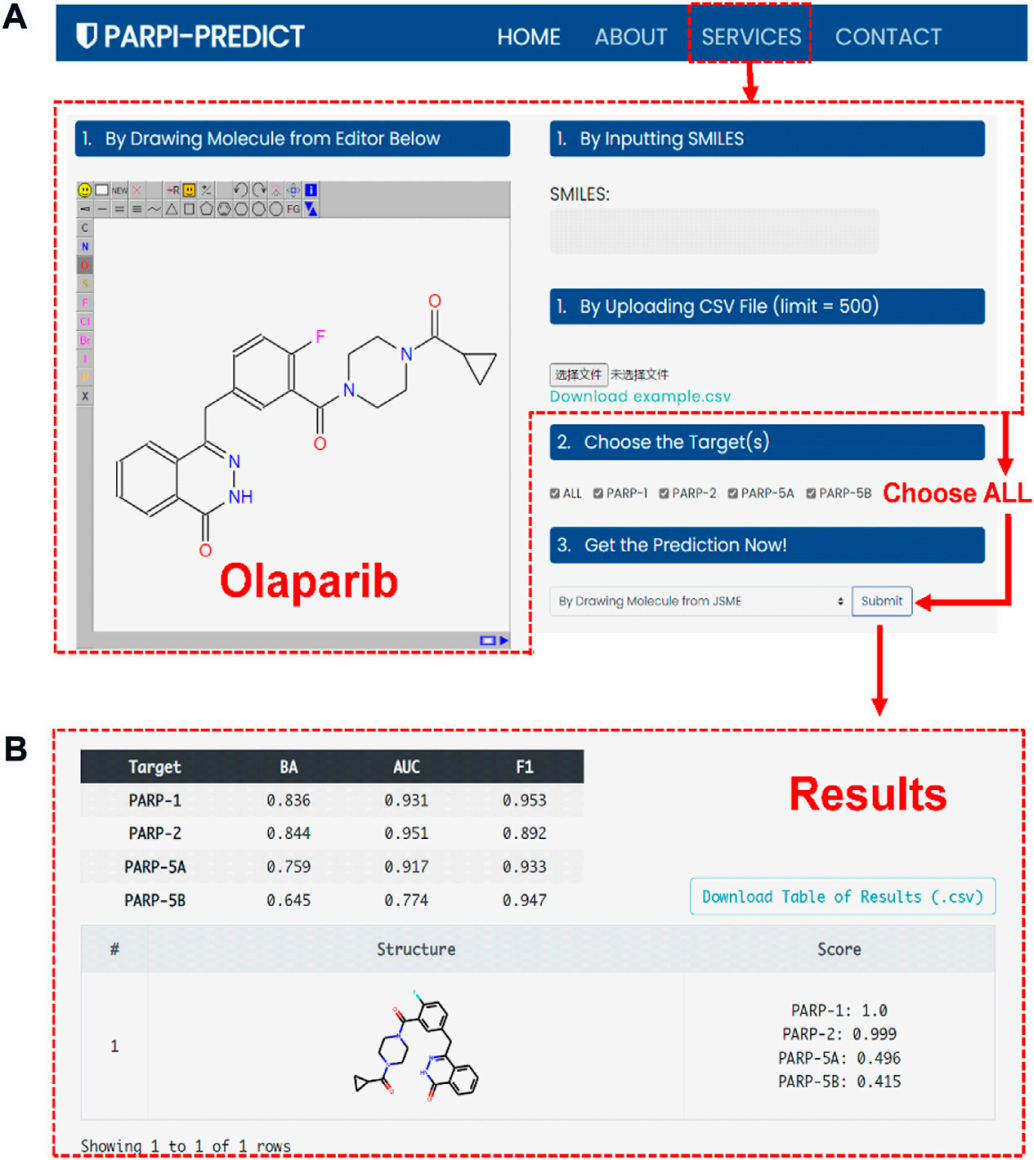
**(A)** The service interface of the PARPi-Predict. **(B)** The prediction results of Olaparib.

To facilitate the design and discovery of novel selective PARP inhibitors by experts or non-experts in the field, an online platform, termed as PARPi-Predict (https://parpipredict.idruglab.cn), was created based on the multi-task FP-GNN model. As shown in Figure 10A, users can draw a structure online, enter or upload multiple molecules in SMILES format to estimate inhibitory activity against PARP of interest or all four isoforms. In addition, a local version executable software (https://github.com/idruglab/PARPi-Predict) was developed to perform large-scale VS.

For example, the predicted scores of olaparib (Figure 10B) were 1.000 and 0.999 for PARP-1 and PARP-2, indicating that olaparib has a powerful inhibitory effect on PARP-1 and PARP-2. Meanwhile, olaparib has the lower predicted scores for PARP-5A (0.496) and PARP-5B (0.415), implying that olaparib may have slightly or no inhibitory activity against PARP-5A and PARP-5B (Figure 10B). Olaparib is an FDA-approved dual PARP-1 (IC_50_ = 0.005 μM) and PARP-2 (IC_50_ = 0.001 μM) inhibitor for the treatment of cancer. Actually, previous study has shown that olaparib exhibits 300-fold greater inhibitory activity against PARP-1 than PARP-5A (IC_50_ ≈ 1.5 μM) (Menear et al., 2008, 1), demonstrating the accuracy and usability of the PARPi-Predict webserver.

## Conclusion

In this work, we first gathered the modelling datasets for four human-derived PARP isoforms, including PARP-1, PARP-2, PARP-5A, and PARP-5B, and then proposed a multi-task FP-GNN model to predict the inhibitory activity of molecules against these four PARP isoforms. Compared with the baseline predictive models, such as 40 fingerprint-based models using ML (i.e., RF, SVM, DNN, XGBoost, and LR) and 28 graph-based DL models (i.e., GAT, GCN, Chemprop, Attentive FP, MPNN, and FP-GNN), the multi-task FP-GNN model achieves the best overall performance on these four PARP isoforms with the highest average values of BA (0.753 ± 0.033), F1 (0.910 ± 0.045), and AUC (0.888 ± 0.016) for the test sets. Y-scrambling testing confirmed that the results of the multi-task FP-GNN model were not random correlations. Additionally, the interpretability of the multi-task FP-GNN model allows researchers to pinpoint critical structural fragments associated with PARP subtype inhibition. Finally, an online platform (called PARPi-Predict) and its local version software were established on the basis of current models, which provide a theoretical foundation for the design and discovery of new selective PARP inhibitors.

In the future, as the number of inhibitors of other PARP subtypes gradually increases, more predictive models will be built and added to the platform for the research community. There are two optimization routes for our future works. On the one hand, pre-trained methods may have great potential due to the insufficient quantity and poor quality of biological datasets including PARPs datasets. Extracting information from a large dataset before training the target dataset can ensure the prescribed minimum of prediction on the target dataset. On the other hand, when training a model on a specific dataset, it is feasible to import the information of the PARP protein into a modified FP-GNN model models and then combine features of molecules and the protein target to predict PARPs inhibitors collectively.

## Data Availability

The original contributions presented in the study are included in the article/Supplementary Material, further inquiries can be directed to the corresponding authors.

## References

[B1] Abbasi-Radmoghaddam Z. Riahi S. Gharaghani S. Mohammadi-Khanaposhtanai M. (2021). Design of potential anti-tumor PARP-1 inhibitors by QSAR and molecular modeling studies. Mol. Divers. 25, 263–277. 10.1007/s11030-020-10063-9 32140890

[B2] Alam S. Khan F. (2019). 3D-QSAR, Docking, ADME/Tox studies on Flavone analogs reveal anticancer activity through Tankyrase inhibition. Sci. Rep. 9, 5414. 10.1038/s41598-019-41984-7 30932078 PMC6443786

[B3] Ali S. Khan F. Galindo-Campos M. Yélamos J. (2016). Understanding specific functions of PARP-2: New lessons for cancer therapy. Am. J. Cancer Res. 6, 1842–1863. 27725894 PMC5043098

[B4] Antolín A. A. Mestres J. (2014). Linking off-target kinase pharmacology to the differential cellular effects observed among PARP inhibitors. Oncotarget 5, 3023–3028. 10.18632/oncotarget.1814 24632590 PMC4102788

[B5] Bai P. Canto C. Brunyánszki A. Huber A. Szántó M. Cen Y. (2011). PARP-2 regulates SIRT1 expression and whole-body energy expenditure. Cell Metab. 13, 450–460. 10.1016/j.cmet.2011.03.013 21459329 PMC3108571

[B6] Bemis G. W. Murcko M. A. (1996). The properties of known drugs. 1. Molecular frameworks. J. Med. Chem. 39, 2887–2893. 10.1021/jm9602928 8709122

[B7] Breiman L. (2001). Random forests. Mach. Learn. 45, 5–32. 10.1023/A:1010933404324

[B8] Cai C. Guo P. Zhou Y. Zhou J. Wang Q. Zhang F. (2019). Deep learning-based prediction of drug-induced cardiotoxicity. J. Chem. Inf. Model. 59, 1073–1084. 10.1021/acs.jcim.8b00769 30715873 PMC6489130

[B9] Cai H. Zhang H. Zhao D. Wu J. Wang L. (2022). FP-GNN: a versatile deep learning architecture for enhanced molecular property prediction. Brief. Bioinform. bbac408. 10.1093/bib/bbac408 36124766

[B10] Chen T. Guestrin C. (2016). “XGBoost: A scalable tree boosting system,” in Proceedings of the 22nd ACM SIGKDD international conference on knowledge discovery and data mining (San Francisco California USA: ACM), 785–794. 10.1145/2939672.2939785

[B11] Cortes C. Vapnik V. (1995). Support-vector networks. Mach. Learn. 20, 273–297. 10.1007/BF00994018

[B12] Curtin N. Bányai K. Thaventhiran J. Le Quesne J. Helyes Z. Bai P. (2020). Repositioning PARP inhibitors for SARS-CoV-2 infection(COVID-19); a new multi-pronged therapy for acute respiratory distress syndrome? Br. J. Pharmacol. 177, 3635–3645. 10.1111/bph.15137 32441764 PMC7280733

[B13] Davis L. J. Offord K. P. (1997). Logistic regression. J. Pers. Assess. 68, 497–507. 10.1207/s15327752jpa6803_3 16372865

[B14] De Murcia J. M. Niedergang C. Trucco C. Ricoul M. Dutrillaux B. Mark M. (1997). Requirement of poly(ADP-ribose) polymerase in recovery from DNA damage in mice and in cells. Proc. Natl. Acad. Sci. U. S. A. 94, 7303–7307. 10.1073/pnas.94.14.7303 9207086 PMC23816

[B15] Dreiseitl S. Ohno-Machado L. (2002). Logistic regression and artificial neural network classification models: a methodology review. J. Biomed. Inf. 35, 352–359. 10.1016/S1532-0464(03)00034-0 12968784

[B16] Durant J. L. Leland B. A. Henry D. R. Nourse J. G. (2002). Reoptimization of MDL keys for use in drug discovery. J. Chem. Inf. Comput. Sci. 42, 1273–1280. 10.1021/ci010132r 12444722

[B17] Durstewitz D. Koppe G. Meyer-Lindenberg A. (2019). Deep neural networks in psychiatry. Mol. Psychiatry 24, 1583–1598. 10.1038/s41380-019-0365-9 30770893

[B18] Duvenaud D. Maclaurin D. Aguilera-Iparraguirre J. Gómez-Bombarelli R. Hirzel T. Aspuru-Guzik A. (2015). “Convolutional networks on graphs for learning molecular fingerprints,” in Proceedings of the 28th International Conference on Neural Information Processing Systems (Cambridge, MA, USA: MIT Press), 2224–2232. NIPS’15.

[B19] Eliasson M. J. L. Sampei K. Mandir A. S. Hurn P. D. Traystman R. J. Bao J. (1997). Poly(ADP-ribose) polymerase gene disruption renders mice resistant to cerebral ischemia. Nat. Med. 3, 1089–1095. 10.1038/nm1097-1089 9334719

[B20] Farmer H. McCabe N. Lord C. J. Tutt A. N. J. Johnson D. A. Richardson T. B. (2005). Targeting the DNA repair defect in BRCA mutant cells as a therapeutic strategy. Nature 434, 917–921. 10.1038/nature03445 15829967

[B21] Ge Y. Tian T. Huang S. Wan F. Li J. Li S. (2021). An integrative drug repositioning framework discovered a potential therapeutic agent targeting COVID-19. Signal Transduct. Target. Ther. 6, 165. 10.1038/s41392-021-00568-6 33895786 PMC8065335

[B22] Gilmer J. Schoenholz S. S. Riley P. F. Vinyals O. Dahl G. E. (2017). Neural message passing for quantum chemistry. ArXiv170401212 Cs. Available at: http://arxiv.org/abs/1704.01212 (Accessed December 28, 2021).

[B23] Guo Q. Zhang H. Deng Y. Zhai S. Jiang Z. Zhu D. (2020). Ligand- and structural-based discovery of potential small molecules that target the colchicine site of tubulin for cancer treatment. Eur. J. Med. Chem. 196, 112328. 10.1016/j.ejmech.2020.112328 32320841

[B24] Hannigan K. Kulkarni S. S. Bdzhola V. G. Golub A. G. Yarmoluk S. M. Talele T. T. (2013). Identification of novel PARP-1 inhibitors by structure-based virtual screening. Bioorg. Med. Chem. Lett. 23, 5790–5794. 10.1016/j.bmcl.2013.09.007 24074844

[B25] He S. Zhao D. Ling Y. Cai H. Cai Y. Zhang J. (2021). Machine learning enables accurate and rapid prediction of active molecules against breast cancer cells. Front. Pharmacol. 12, 796534. Available at: https://www.frontiersin.org/article/10.3389/fphar.2021.796534 . 10.3389/fphar.2021.796534 34975493 PMC8719637

[B26] Horvath D. Marcou G. Varnek A. (2010). A unified approach to the applicability domain problem of QSAR models. J. Cheminform. 2, O6. 10.1186/1758-2946-2-S1-O6

[B27] Hsiao S. J. Smith S. (2008). Tankyrase function at telomeres, spindle poles, and beyond. Biochimie 90, 83–92. 10.1016/j.biochi.2007.07.012 17825467

[B28] Karlberg T. Markova N. Johansson I. Hammarström M. Schütz P. Weigelt J. (2010). Structural basis for the interaction between tankyrase-2 and a potent wnt-signaling inhibitor. J. Med. Chem. 53, 5352–5355. 10.1021/jm100249w 20565110

[B29] Kim S. Chen J. Cheng T. Gindulyte A. He J. He S. (2020). PubChem in 2021: new data content and improved web interfaces. Nucleic Acids Res. 49, D1388–D1395. 10.1093/nar/gkaa971 PMC7778930 33151290

[B30] Kim D.-S. Camacho C. V. Kraus W. L. (2021). Alternate therapeutic pathways for PARP inhibitors and potential mechanisms of resistance. Exp. Mol. Med. 53, 42–51. 10.1038/s12276-021-00557-3 33487630 PMC8080675

[B31] Kinosada H. Okada-Iwasaki R. Kunieda K. Suzuki-Imaizumi M. Takahashi Y. Miyagi H. (2021). The dual pocket binding novel tankyrase inhibitor K-476 enhances the efficacy of immune checkpoint inhibitor by attracting CD8+ T cells to tumors. Am. J. Cancer Res. 11, 264–276. 33520373 PMC7840722

[B32] Kirubakaran P. Arunkumar P. Premkumar K. Muthusamy K. (2014). Sighting of tankyrase inhibitors by structure- and ligand-based screening and *in vitro* approach. Mol. Biosyst. 10, 2699–2712. 10.1039/C4MB00309H 25091558

[B33] LaFargue C. J. Dal Molin G. Z. Sood A. K. Coleman R. L. (2019). Exploring and comparing adverse events between PARP inhibitors. Lancet. Oncol. 20, e15–e28. 10.1016/S1470-2045(18)30786-1 30614472 PMC7292736

[B34] Li H. Liu Z.-Y. Wu N. Chen Y.-C. Cheng Q. Wang J. (2020). PARP inhibitor resistance: the underlying mechanisms and clinical implications. Mol. Cancer 19, 107. 10.1186/s12943-020-01227-0 32563252 PMC7305609

[B35] Liu T. Lin Y. Wen X. Jorissen R. N. Gilson M. K. (2007). BindingDB: a web-accessible database of experimentally determined protein–ligand binding affinities. Nucleic Acids Res. 35, D198–D201. 10.1093/nar/gkl999 17145705 PMC1751547

[B36] Lucarini L. Durante M. Lanzi C. Pini A. Boccalini G. Calosi L. (2017). HYDAMTIQ, a selective PARP-1 inhibitor, improves bleomycin-induced lung fibrosis by dampening the TGF-β/SMAD signalling pathway. J. Cell. Mol. Med. 21, 324–335. 10.1111/jcmm.12967 27704718 PMC5264150

[B37] Luo Y. Zeng R. Guo Q. Xu J. Sun X. Wang L. (2019). Identifying a novel anticancer agent with microtubule-stabilizing effects through computational cell-based bioactivity prediction models and bioassays. Org. Biomol. Chem. 17, 1519–1530. 10.1039/c8ob02193g 30681116

[B38] Masutani M. Suzuki H. Kamada N. Watanabe M. Ueda O. Nozaki T. (1999). Poly(ADP-ribose) polymerase gene disruption conferred mice resistant to streptozotocin-induced diabetes. Proc. Natl. Acad. Sci. U. S. A. 96, 2301–2304. 10.1073/pnas.96.5.2301 10051636 PMC26778

[B39] Mateo J. Lord C. J. Serra V. Tutt A. Balmana J. Castroviejo-Bermejo M. (2019). A decade of clinical development of PARP inhibitors in perspective. Ann. Oncol. 30, 1437–1447. 10.1093/annonc/mdz192 31218365 PMC6771225

[B40] Mendez D. Gaulton A. Bento A. P. Chambers J. De Veij M. Félix E. (2019). ChEMBL: towards direct deposition of bioassay data. Nucleic Acids Res. 47, D930–D940. 10.1093/nar/gky1075 30398643 PMC6323927

[B41] Menear K. A. Adcock C. Boulter R. Cockcroft X. Copsey L. Cranston A. (2008). 4-[3-(4-Cyclopropanecarbonylpiperazine-1-carbonyl)-4-fluorobenzyl]-2 *H* -phthalazin-1-one: A novel bioavailable inhibitor of poly(ADP-ribose) polymerase-1. J. Med. Chem. 51, 6581–6591. 10.1021/jm8001263 18800822

[B42] Nguyen-Vo T.-H. Trinh Q. H. Nguyen L. Nguyen-Hoang P.-U. Nguyen T.-N. Nguyen D. T. (2021). iCYP-MFE: Identifying human cytochrome P450 inhibitors using multitask learning and molecular fingerprint-embedded encoding. J. Chem. Inf. Model. 10.1021/acs.jcim.1c00628 34672553

[B43] Papeo G. Posteri H. Borghi D. Busel A. A. Caprera F. Casale E. (2015). Discovery of 2-[1-(4, 4-Difluorocyclohexyl)piperidin-4-yl]-6-fluoro-3-oxo-2, 3-dihydro-1H-isoindole-4-carboxamide (NMS-P118): A potent, orally available, and highly selective PARP-1 inhibitor for cancer therapy. J. Med. Chem. 58, 6875–6898. 10.1021/acs.jmedchem.5b00680 26222319

[B44] Pedregosa F. Varoquaux G. Gramfort A. Michel V. Thirion B. Grisel O. (2018). Scikit-learn: Machine learning in Python. ArXiv12010490 Cs. Available at: http://arxiv.org/abs/1201.0490 (Accessed December 27, 2021).

[B45] Petras D. Caraballo-Rodríguez A. M. Jarmusch A. K. Molina-Santiago C. Gauglitz J. M. Gentry E. C. (2021). Chemical proportionality within molecular networks. Anal. Chem. 93, 12833–12839. 10.1021/acs.analchem.1c01520 34533933

[B46] Riahi S. Pourbasheer E. Dinarvand R. Ganjali M. R. Norouzi P. (2008). QSAR study of 2-(1-Propylpiperidin-4-yl)-1H-Benzimidazole-4-Carboxamide as PARP inhibitors for treatment of cancer. Chem. Biol. Drug Des. 72, 575–584. 10.1111/j.1747-0285.2008.00739.x 19090924

[B47] Rogers D. Hahn M. (2010). Extended-connectivity fingerprints. J. Chem. Inf. Model. 50, 742–754. 10.1021/ci100050t 20426451

[B48] Sharma M. C. (2016). Structural requirements of some 2-(1-Propylpiperidin-4-yl)-1H-benzimidazole-4-carboxamide derivatives as poly (ADP-Ribose) polymerase (PARP) for the treatment of cancer: QSAR approach. Interdiscip. Sci. 8, 11–22. 10.1007/s12539-015-0015-0 26205198

[B49] Shirai F. Tsumura T. Yashiroda Y. Yuki H. Niwa H. Sato S. (2019). Discovery of novel spiroindoline derivatives as selective tankyrase inhibitors. J. Med. Chem. 62, 3407–3427. 10.1021/acs.jmedchem.8b01888 30883102

[B50] Shultz M. D. Kirby C. A. Stams T. Chin D. N. Blank J. Charlat O. (2012). [1, 2, 4]Triazol-3-ylsulfanylmethyl)-3-phenyl-[1, 2, 4]oxadiazoles: Antagonists of the wnt pathway that inhibit tankyrases 1 and 2 via novel adenosine pocket binding. J. Med. Chem. 55, 1127–1136. 10.1021/jm2011222 22260203

[B51] Shultz M. D. Cheung A. K. Kirby C. A. Firestone B. Fan J. Chen C. H.-T. (2013). Identification of NVP-TNKS656: The use of structure–efficiency relationships to generate a highly potent, selective, and orally active tankyrase inhibitor. J. Med. Chem. 56, 6495–6511. 10.1021/jm400807n 23844574

[B52] Stiefl N. Watson I. A. Baumann K. Zaliani A. (2006). ErG: 2D pharmacophore descriptions for scaffold hopping. J. Chem. Inf. Model. 46, 208–220. 10.1021/ci050457y 16426057

[B53] Stoica B. A. Loane D. J. Zhao Z. Kabadi S. V. Hanscom M. Byrnes K. R. (2014). PARP-1 inhibition attenuates neuronal loss, microglia activation and neurological deficits after traumatic brain injury. J. Neurotrauma 31, 758–772. 10.1089/neu.2013.3194 24476502 PMC3967421

[B54] Szabo C. Martins V. Liaudet L. (2020). Poly(ADP-Ribose) polymerase inhibition in acute lung injury. A reemerging concept. Am. J. Respir. Cell Mol. Biol. 63, 571–590. 10.1165/rcmb.2020-0188TR 32640172 PMC7605157

[B55] Tomassi S. Pfahler J. Mautone N. Rovere A. Esposito C. Passeri D. (2020). From PARP1 to TNKS2 inhibition: A structure-based approach. ACS Med. Chem. Lett. 11, 862–868. 10.1021/acsmedchemlett.9b00654 32435397 PMC7236224

[B56] Veličković P. Cucurull G. Casanova A. Romero A. Liò P. Bengio Y. (2018). Graph attention networks. ArXiv171010903 Cs Stat. Available at: http://arxiv.org/abs/1710.10903 (Accessed December 28, 2021).

[B57] Wang G. Huang X. Li Y. Guo K. Ning P. Zhang Y. (2013). PARP-1 inhibitor, DPQ, attenuates LPS-induced acute lung injury through inhibiting NF-κB-Mediated inflammatory response. PLoS ONE 8, e79757. 10.1371/journal.pone.0079757 24278171 PMC3836796

[B58] Wang L. Le X. Li L. Ju Y. Lin Z. Gu Q. (2014). Discovering new agents active against methicillin-resistant *Staphylococcus aureus* with ligand-based approaches. J. Chem. Inf. Model. 54, 3186–3197. 10.1021/ci500253q 25375651

[B59] Wang L. Chen L. Yu M. Xu L. H. Cheng B. Lin Y. S. (2016a). Discovering new mTOR inhibitors for cancer treatment through virtual screening methods and *in vitro* assays. Sci. Rep. 6, 18987. 10.1038/srep18987 26732172 PMC4702177

[B60] Wang L. Li Y. Xu M. Pang X. Liu Z. Tan W. (2016b). Chemical fragment-based CDK4/6 inhibitors prediction and web server. RSC Adv. 6, 16972–16981. 10.1039/C5RA23289A

[B61] Wang L. Pang X. Li Y. Zhang Z. Tan W. (2017). RADER: a RApid DEcoy retriever to facilitate decoy based assessment of virtual screening. Bioinformatics 33, 1235–1237. 10.1093/bioinformatics/btw783 28011765

[B62] Wang C. Xu W. An J. Liang M. Li Y. Zhang F. (2019). Poly(ADP-ribose) polymerase 1 accelerates vascular calcification by upregulating Runx2. Nat. Commun. 10, 1203. 10.1038/s41467-019-09174-1 30867423 PMC6416341

[B63] Wu Z. Ramsundar B. Feinberg E. N. Gomes J. Geniesse C. Pappu A. S. (2017). MoleculeNet: A benchmark for molecular machine learning. ArXiv170300564 Phys. Stat. Available at: http://arxiv.org/abs/1703.00564 (Accessed May 7, 2022). 10.1039/c7sc02664a PMC5868307 29629118

[B64] Xiong Z. Wang D. Liu X. Zhong F. Wan X. Li X. (2020). Pushing the boundaries of molecular representation for drug discovery with the graph attention mechanism. J. Med. Chem. 63, 8749–8760. 10.1021/acs.jmedchem.9b00959 31408336

[B65] Yang K. Swanson K. Jin W. Coley C. Eiden P. Gao H. (2019). Analyzing learned molecular representations for property prediction. J. Chem. Inf. Model. 59, 3370–3388. 10.1021/acs.jcim.9b00237 31361484 PMC6727618

[B66] Zeng H. Zhang H. Jang F. Zhao L. Zhang J. (2011). Molecular modeling studies on benzimidazole carboxamide derivatives as PARP-1 inhibitors using 3D-QSAR and docking. Chem. Biol. Drug Des. 78, 333–352. 10.1111/j.1747-0285.2011.01139.x 21585709

[B67] Zheng L. Ren R. Sun X. Zou Y. Shi Y. Di B. (2021). Discovery of a dual tubulin and poly(ADP-ribose) polymerase-1 inhibitor by structure-based pharmacophore modeling, virtual screening, molecular docking, and biological evaluation. J. Med. Chem. 64, 15702–15715. 10.1021/acs.jmedchem.1c00932 34670362

[B68] Zingarelli B. Salzman A. L. Szabó C. (1998). Genetic disruption of poly (ADP-Ribose) synthetase inhibits the expression of P-selectin and intercellular adhesion molecule-1 in myocardial ischemia/reperfusion injury. Circ. Res. 83, 85–94. 10.1161/01.RES.83.1.85 9670921

