# Supplementary material for: A multi-task FP-GNN framework enables accurate prediction of selective PARP inhibitors
Source: Front Pharmacol. 2022 Oct 11;13:971369. doi: 10.3389/fphar.2022.971369 (PMC9592829; doi:10.3389/fphar.2022.971369)
Supplement: Supplementary file 1 [file DataSheet1.docx]

Supplementary Material

**A multi-task FP-GNN framework enables accurate prediction of selective PARP inhibitors**

Daiqiao Ai,^1,‡^ Jingxing Wu,^1,‡^ Hanxuan Cai^1^, Duancheng Zhao^1^, Yihao Chen^1^, Jiajia Wei^1^, Jianrong Xu^3,4^, Jiquan Zhang^2,*^ and Ling Wang^1,*^

*^1^Guangdong Provincial Key Laboratory of Fermentation and Enzyme Engineering, Joint International Research Laboratory of Synthetic Biology and Medicine, Guangdong Provincial Engineering and Technology Research Center of Biopharmaceuticals, School of Biology and Biological Engineering, South China University of Technology, Guangzhou 510006, China.*

*^2^ Guizhou Provincial Engineering Technology Research Center for Chemical Drug R&D, College of Pharmacy, Guizhou Medical University, Guiyang, 550025, China.*

*^3^Department of Pharmacology and Chemical Biology, Shanghai Jiao Tong University School of Medicine, Shanghai, 200025, China.*

*^4^Academy of Integrative Medicine, Shanghai University of Traditional Chinese Medicine, Shanghai 201203, China.*

^‡^These authors contributed equally to this work

*To whom correspondence should be addressed.

**Contents**

**Supplementary Figure S1.** Performance of fingerprint-based ML models. (A) and (B) represent the BA, F1, and AUC values of Morgan-based and MACCS-based predictive models, respectively.

**Supplementary Figure S2.** Y-scrambling results of the multi-task FP-GNN model for PARP-1 (A), PARP-2 (B), PARP-5A (C), and PARP-5B (D). Both the training sets and testing sets were unscrambled (gold). The training sets were scrambled, whereas the test sets were unscrambled (green). The training sets were unscrambled, whereas the test sets were scrambled (purple).

**Supplementary Figure S3.** The predicted 3D binding modes of CHEMBL2419697 to (A) PARP-5A (PDB ID: 3UDD) and (B) PARP-5B (PDB ID: 7CE4).

**Supplementary Figure S4.** The predicted binding modes of the molecule (CHEMBL2419697) to PARP-1 (PDB ID: 5DS3) (Glide-XP docking score: −4.524 kcal/mol) (A) and PARP-2 (PDB ID: 4ZZY) (Glide-XP docking score: −3.810 kcal/mol) (B). The binding modes were predicted using Glide-XP docking.

**Supplementary Figure S5.** The active molecule structure (CHEMBL2419697) and the corresponding bits identified with (ErG) feature separation. D: H-bond donor; Ac: H-bond acceptor; Hf: hydrophobic group; Ar: aromatic ring system.

**Supplementary Figure S6.** The importance of molecular fingerprints during the prediction process. The substructures represented by the 4th, 5th, 12th, 13th, 14th, and 19th bits are important components of the active molecule.

**Supplementary Table S1.** The number of active and inactive compounds in the training, validation, and test sets. (Supplementary Table.xlsx)

**Supplementary Table S2.** Summary of hyperparameter setting in different models. (Supplementary Table.xlsx)

**Supplementary Table S3.** The performance results of models based on Morgan fingerprints. (Supplementary Table.xlsx)

**Supplementary Table S4.** The performance results of models based on MACCS keys. (Supplementary Table.xlsx)

**Supplementary Table S5.** The optimal fingerprint-based ML model for each PARP isoform (sorted by BA value).

**Supplementary Table S6.** The performance results of models based on molecular graph. (Supplementary Table.xlsx)

**Supplementary Table S7.** The performance results of FP-GNN and multi-task FP-GNN models. (Supplementary Table.xlsx)

**Supplementary Table S8.** Average values for ACC, F1, BA, SE, SP, MCC, and AUC of all prediction models (sorted by BA value). (Supplementary Table.xlsx)

**Supplementary Table S9.** The detailed results of redocking studies. (Supplementary Table.xlsx)

**Supplementary Table S10.** The twenty most significant bits of the mixed fingerprints on the prediction of the PARP datasets. (Supplementary Table.xlsx)

**Supplementary Table S11.** The amounts of compounds outside the applicability domain in the test set at different *Z* and k values. (Supplementary Table.xlsx)

**Supplementary Table S12.** The evaluation performance of compounds both inside domain (ID) and outside the domain (OD) in the test set at different *Z* and k values. (Supplementary Table.xlsx)


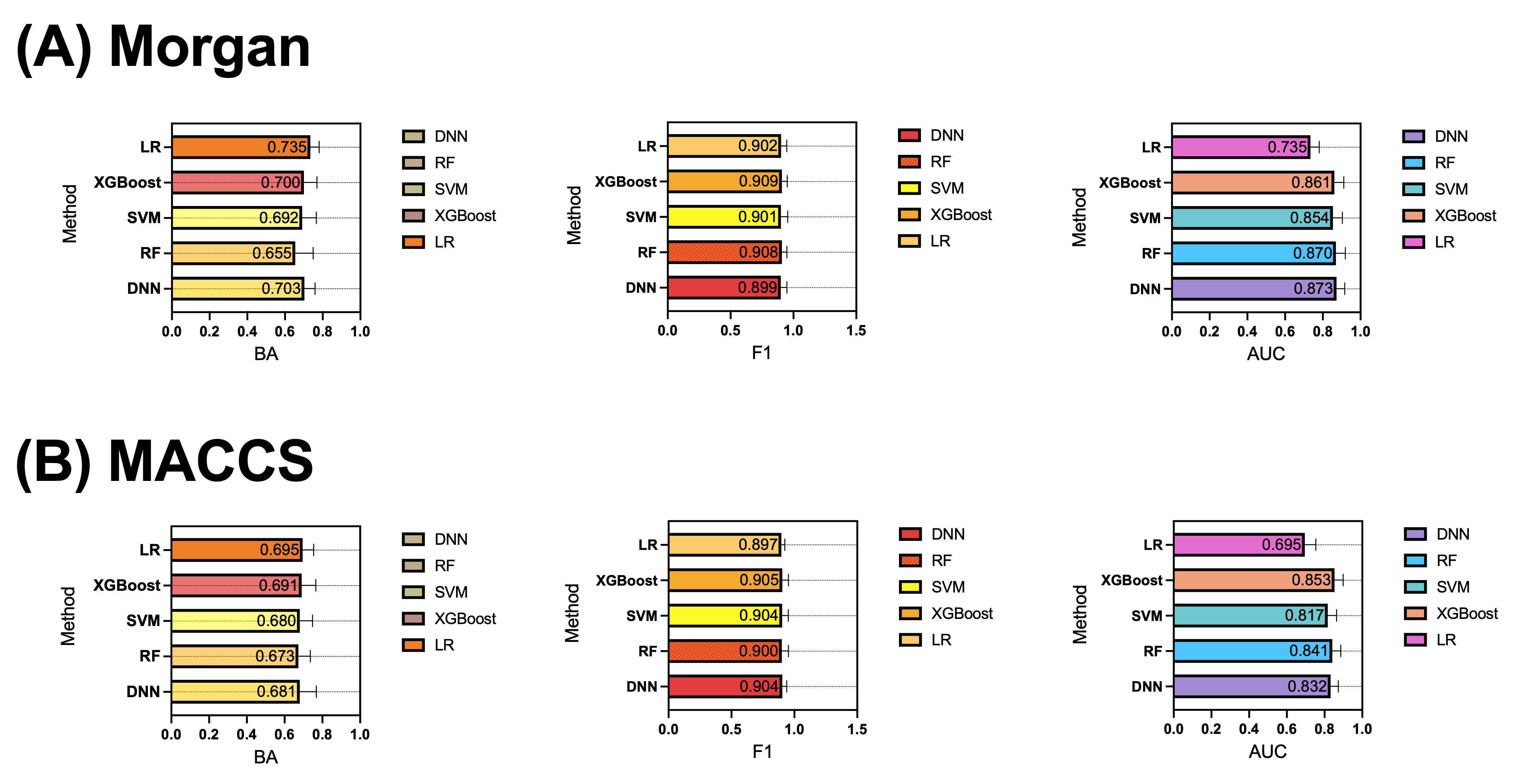


**Supplementary Figure S1.** Performance of fingerprint-based ML models. (A) and (B) represent the BA, F1, and AUC values of Morgan-based and MACCS-based predictive models, respectively.


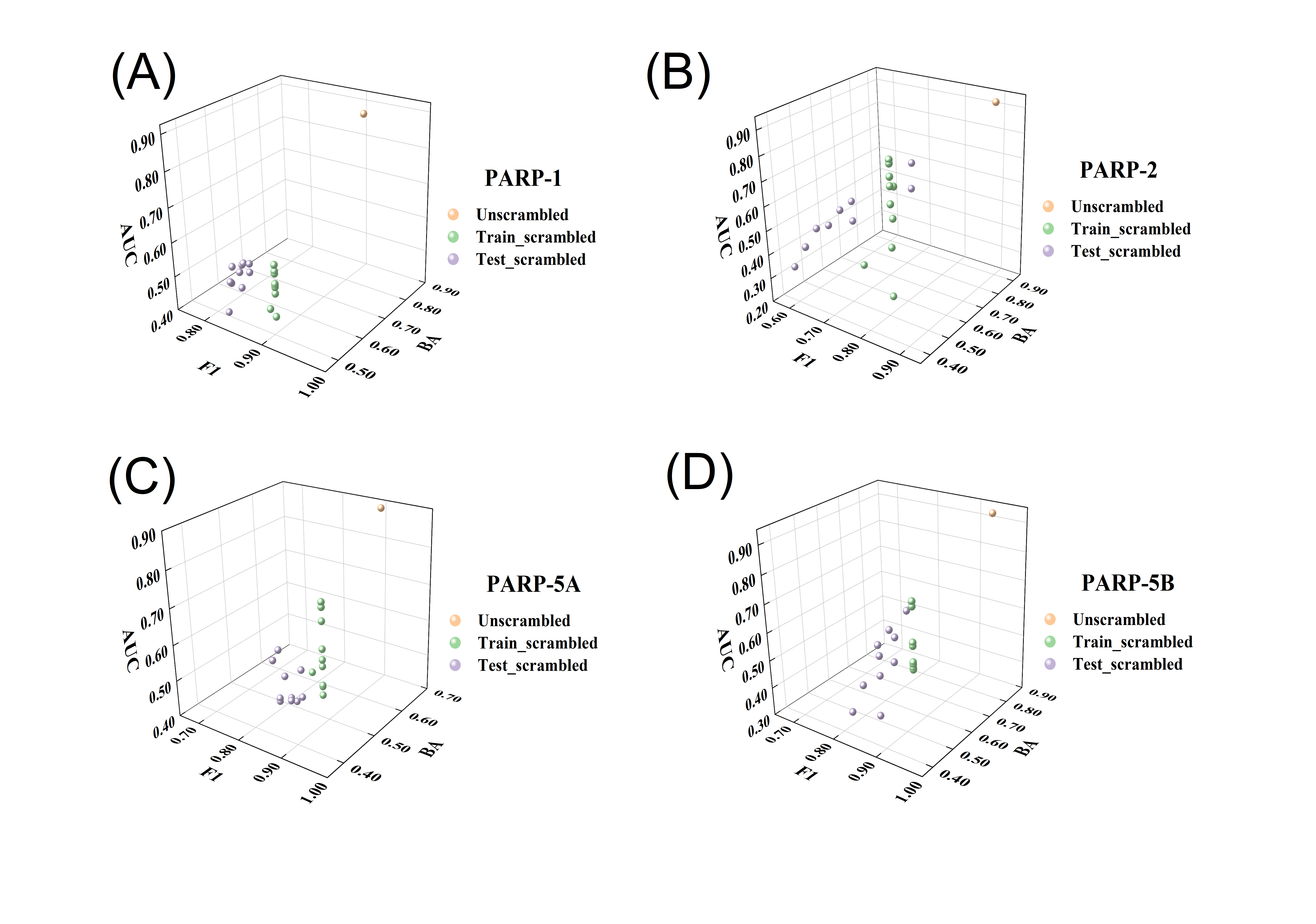


**Supplementary Figure S2.** Y-scrambling results of the multi-task FP-GNN model for PARP-1 (A), PARP-2 (B), PARP-5A (C), and PARP-5B (D). Both the training sets and testing sets were unscrambled (gold). The training sets were scrambled, whereas the test sets were unscrambled (green). The training sets were unscrambled, whereas the test sets were scrambled (purple).


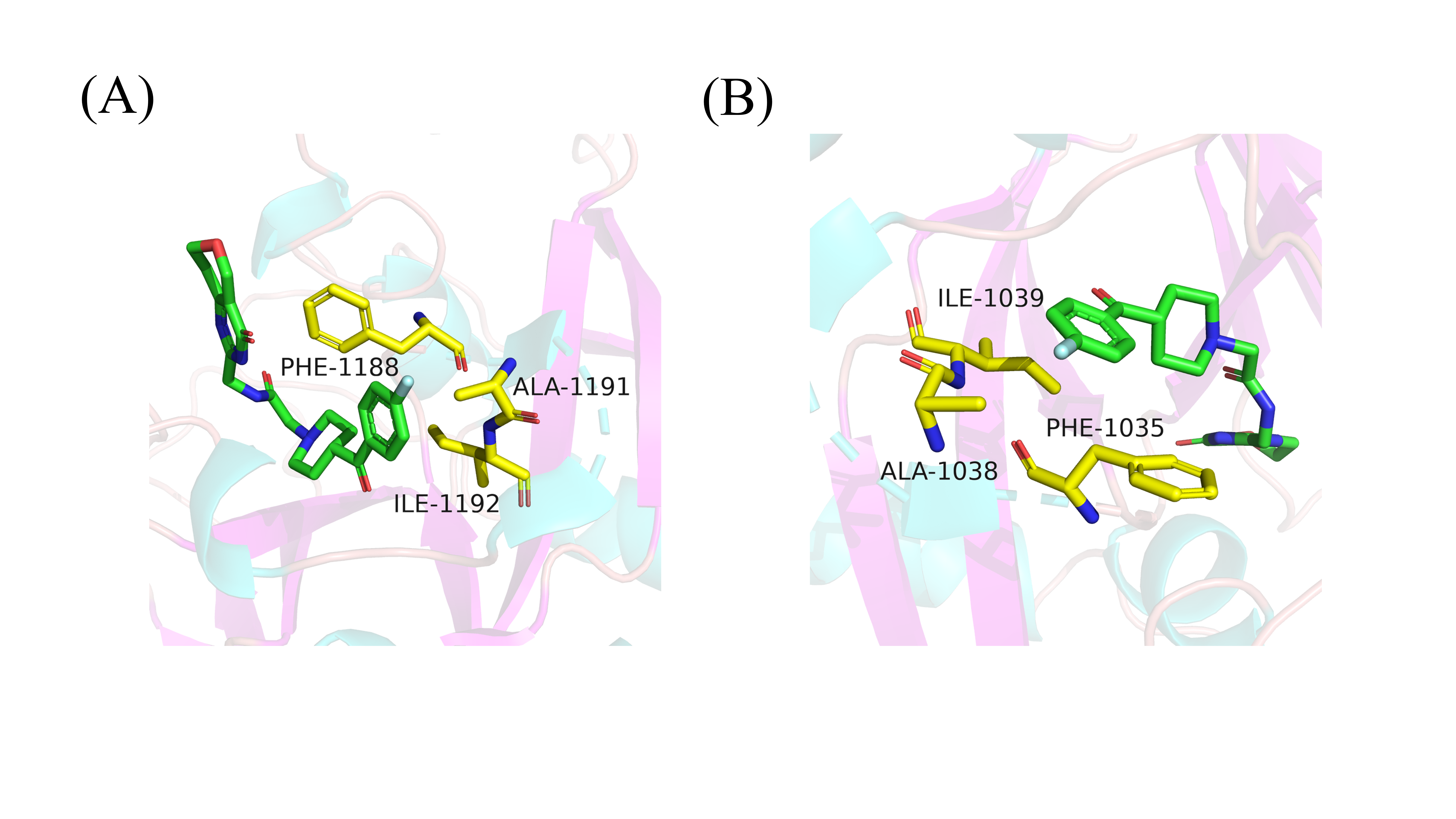


**Supplementary Figure S3.** The predicted 3D binding modes of CHEMBL2419697 to (A) PARP-5A (PDB ID: 3UDD) and (B) PARP-5B (PDB ID: 7CE4).

**
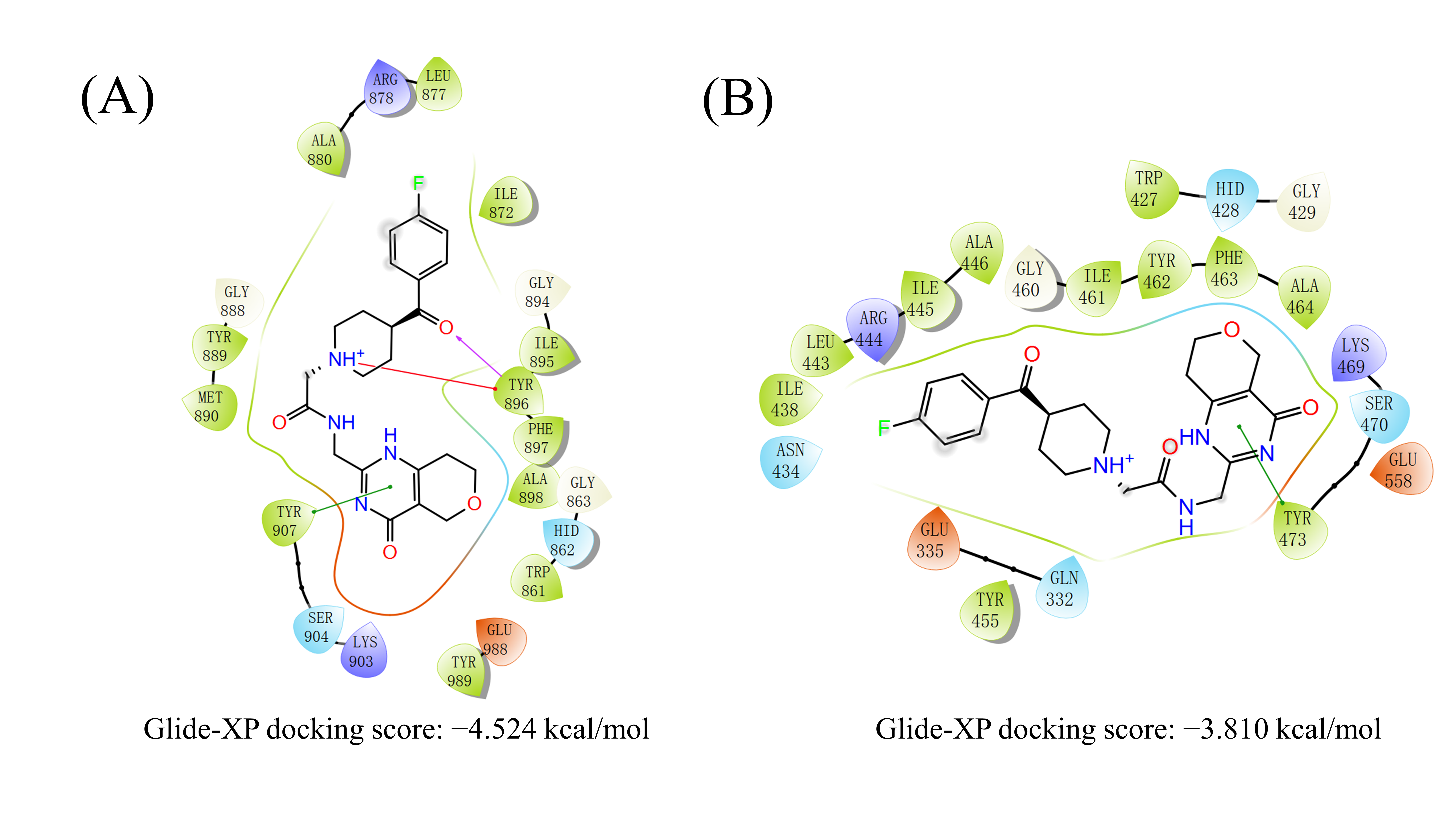
**

**Supplementary Figure S4.** The predicted binding modes of the molecule (CHEMBL2419697) to PARP-1 (PDB ID: 5DS3) (Glide-XP docking score: −4.524 kcal/mol) (A) and PARP-2 (PDB ID: 4ZZY) (Glide-XP docking score: −3.810 kcal/mol) (B). The binding modes were predicted using Glide-XP docking.

**
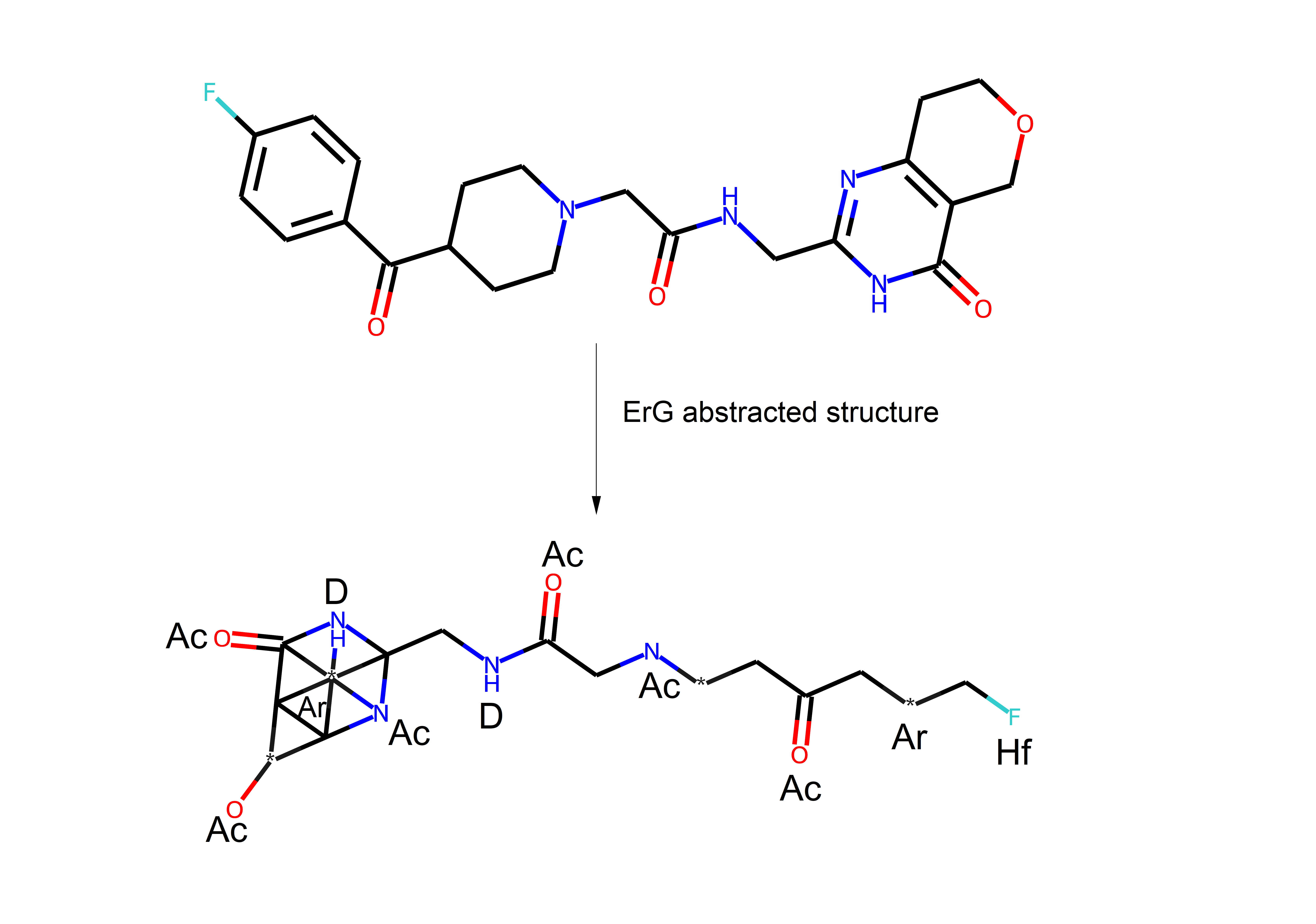
**

**Supplementary Figure S5.** The active molecule structure (CHEMBL2419697) and the corresponding bits identified with (ErG) feature separation. D: H-bond donor; Ac: H-bond acceptor; Hf: hydrophobic group; Ar: aromatic ring system.

**
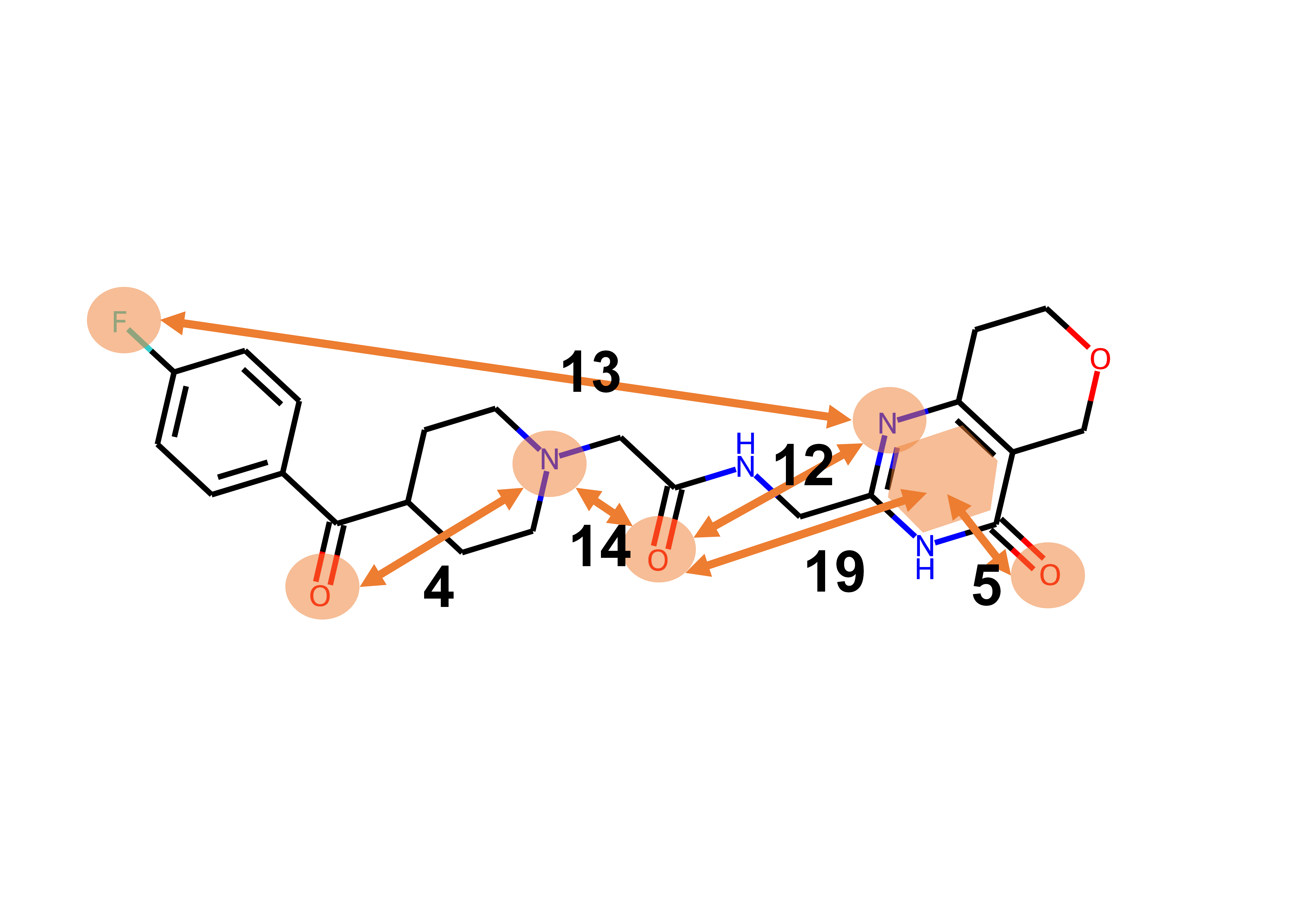
**

**Supplementary Figure S6.** The importance of molecular fingerprints during the prediction process. The substructures represented by the 4th, 5th, 12th, 13th, 14th, and 19th bits are important components of the active molecule.
